# Analysis of five deep-sequenced trio-genomes of the Peninsular Malaysia Orang Asli and North Borneo populations

**DOI:** 10.1186/s12864-019-6226-8

**Published:** 2019-11-12

**Authors:** Lian Deng, Haiyi Lou, Xiaoxi Zhang, Bhooma Thiruvahindrapuram, Dongsheng Lu, Christian R. Marshall, Chang Liu, Bo Xie, Wanxing Xu, Lai-Ping Wong, Chee-Wei Yew, Aghakhanian Farhang, Rick Twee-Hee Ong, Mohammad Zahirul Hoque, Abdul Rahman Thuhairah, Bhak Jong, Maude E. Phipps, Stephen W. Scherer, Yik-Ying Teo, Subbiah Vijay Kumar, Boon-Peng Hoh, Shuhua Xu

**Affiliations:** 1 Key Laboratory of Computational Biology, CAS-MPG Partner Institute for Computational Biology, Shanghai Institute of Nutrition and Health, Shanghai Institutes for Biological Sciences, University of Chinese Academy of Sciences, Chinese Academy of Sciences, Shanghai, China; 2 School of Life Science and Technology, ShanghaiTech University, Shanghai, 201210 China; 3 The Centre for Applied Genomics, The Hospital for Sick Children, Toronto, Ontario Canada; 4 Genome Diagnostics, Department of Paediatric Laboratory Medicine, The Hospital for Sick Children, Toronto, Ontario Canada; 5 Laboratory Medicine and Pathobiology, University of Toronto, Toronto, Ontario Canada; 6 Saw Swee Hock School of Public Health, National University of Singapore, Singapore, 117597 Singapore; 7 Biotechnology Research Institute, Universiti Malaysia Sabah, Jalan UMS, 88400 Kota Kinabalu, Sabah Malaysia; 8 Jefrey Cheah School of Medicine and Health Sciences, Monash University Malaysia, Jalan Lagoon Selatan, Sunway, 46150 Subang Jaya, Selangor Malaysia; 9 Tropical Medicine and Biology Platform, Monash University Malaysia, Jalan Lagoon Selatan, 46150 Sunway, Subang Jaya, Selangor Malaysia; 10 Faculty of Medicine and Health Sciences, Universiti Malaysia Sabah, Jalan UMS, 88400 Kota Kinabalu, Sabah Malaysia; 11 Clinical Pathology Diagnostic Centre Research Laboratory, Faculty of Medicine, Universiti Teknologi MARA, Sungai Buloh Campus, 47000 Sg Buloh, Subang Jaya, Selangor Malaysia; 12 Personal Genomics Institute, Genome Research Foundation, Suwon, Republic of Korea; 13 Geromics, Ulsan, 44919 Republic of Korea; 14 Biomedical Engineering Department, The Genomics Institute, UNIST, Ulsan, Republic of Korea; 15 Program in Genetics and Genome Biology, The Hospital for Sick Children, Toronto, Ontario Canada; 16 Department of Molecular Genetics, University of Toronto, Toronto, Ontario Canada; 17 NUS Graduate School for Integrative Science and Engineering, National University of Singapore, Singapore, 117456 Singapore; 18 Life Sciences Institute, National University of Singapore, Singapore, Singapore; 19 Department of Statistics and Applied Probability, National University of Singapore, Singapore, Singapore; 20 Genome Institute of Singapore, Agency for Science, Technology and Research, Singapore, 138672 Singapore; 21 Faculty of Medicine and Health Sciences, UCSI University, Jalan Menara Gading, Taman Connaught, Cheras, 56000 Kuala Lumpur, Malaysia; 22 Center for Excellence in Animal Evolution and Genetics, Chinese Academy of Sciences, Kunming, 650223 China; 23 Collaborative Innovation Center of Genetics and Development, Shanghai, 200438 China; 24 Human Phenome Institute, Fudan University, Shanghai, 201203 China

## Abstract

**Background:**

Recent advances in genomic technologies have facilitated genome-wide investigation of human genetic variations. However, most efforts have focused on the major populations, yet trio genomes of indigenous populations from Southeast Asia have been under-investigated.

**Results:**

We analyzed the whole-genome deep sequencing data (~ 30×) of five native trios from Peninsular Malaysia and North Borneo, and characterized the genomic variants, including single nucleotide variants (SNVs), small insertions and deletions (indels) and copy number variants (CNVs). We discovered approximately 6.9 million SNVs, 1.2 million indels, and 9000 CNVs in the 15 samples, of which 2.7% SNVs, 2.3% indels and 22% CNVs were novel, implying the insufficient coverage of population diversity in existing databases. We identified a higher proportion of novel variants in the Orang Asli (OA) samples, i.e., the indigenous people from Peninsular Malaysia, than that of the North Bornean (NB) samples, likely due to more complex demographic history and long-time isolation of the OA groups. We used the pedigree information to identify de novo variants and estimated the autosomal mutation rates to be 0.81 × 10^− 8^ – 1.33 × 10^− 8^, 1.0 × 10^− 9^ – 2.9 × 10^− 9^, and ~ 0.001 per site per generation for SNVs, indels, and CNVs, respectively. The trio-genomes also allowed for haplotype phasing with high accuracy, which serves as references to the future genomic studies of OA and NB populations. In addition, high-frequency inherited CNVs specific to OA or NB were identified. One example is a 50-kb duplication in *DEFA1B* detected only in the Negrito trios, implying plausible effects on host defense against the exposure of diverse microbial in tropical rainforest environment of these hunter-gatherers. The CNVs shared between OA and NB groups were much fewer than those specific to each group. Nevertheless, we identified a 142-kb duplication in *AMY1A* in all the 15 samples, and this gene is associated with the high-starch diet. Moreover, novel insertions shared with archaic hominids were identified in our samples.

**Conclusion:**

Our study presents a full catalogue of the genome variants of the native Malaysian populations, which is a complement of the genome diversity in Southeast Asians. It implies specific population history of the native inhabitants, and demonstrated the necessity of more genome sequencing efforts on the multi-ethnic native groups of Malaysia and Southeast Asia.

## Background

The rapid development of genome sequencing technology and analysis capabilities has spawned large scale human genome sequencing projects in recent years, for instance, the 1000 Genomes Project, the Simons Genome Diversity Project, the Estonian Biocentre Human Genome Diversity Project, UK10K Project, the All of Us Research Program (https://allofus.nih.gov/), and others [1–4]. A major undertaking of these projects is to conduct a comprehensive inventory of all detectable variations of global modern human populations, which is important for characterizing the human genetic diversity as well as identifying disease risk variants. The fine-scale analyses of the human genome require accurate identification of variants, imputation and phasing of genotypes, which may be greatly facilitated by increasing the sequencing depth and using pedigree information, especially for genomic regions containing large and complex variations like structural variants (SVs) and small insertions and deletions (indels) [5]. In addition, the trio information allows verification of the detected variants using Mendel’s law of inheritance and detecting de novo mutations. Understanding the rates and patterns of de novo mutations is important for analyzing the population relationship [6, 7], detecting natural selection [8, 9], and mapping genes underlying complex traits [10]. To date, most trio-based sequencing studies are disease-related [11–13]. Whole-genome sequencing studies of healthy trios are less biased than those of the disease-based ones, but publications on these are rather limited, except for the one Vietnamese trio and 10 Danish trios that were sequenced to high coverage in recent years [14, 15].

Located at the crossroads of Southeast Asia, Malaysia is rich with human population diversity, including native Malays and Orang Asli (OA, a collective term of indigenous populations) occupying the Peninsular Malaysia, and over 40 native ethnic groups categorized based on linguistic and socio-economy practices in North Borneo [16]. However, these native populations are largely underrepresented in the whole-genome sequencing projects. The genomic architecture of these populations were characterized by a handful of SNP-array-based genome-wide studies [17–22]. Recently, using the whole genome sequencing data of 12 unrelated individuals, we have also revealed the population structure and divergence between native populations from Peninsular Malaysia and North Borneo [23].

In this study, we present the variant catalogue of five native trios (father-mother-offspring) from Peninsular Malaysia (OA, including Bateq, Mendriq and Semai) and North Borneo (NB, including Dusun and Murut) by whole-genome sequencing to a mean depth of 30×. Our data revealed a large number of novel genomic variants, including the single nucleotide variants (SNVs), indels and copy number variants (CNVs), in the native Malaysian trios, particularly in OA. The rates of de novo genomic variants were estimated. In addition, the inherited novel insertions were identified from the unmapped reads of these samples, some of which could have been shared with archaic hominins.

## Results

### Discovery of SNVs and indels

**Fig. 1:**
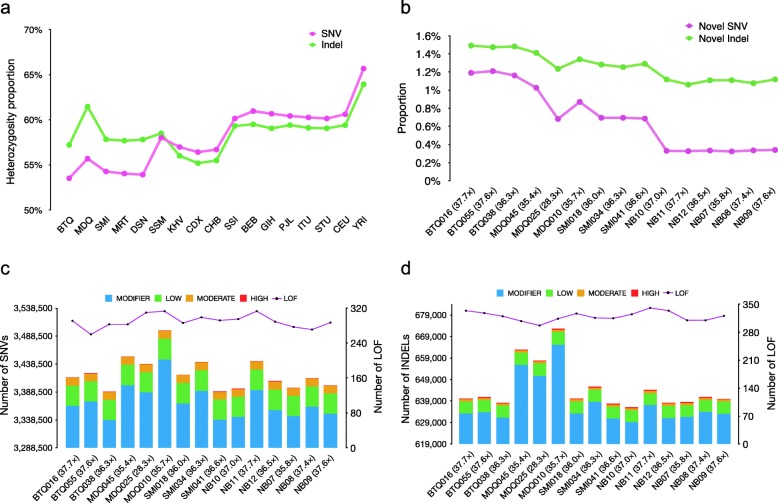
Characterization of SNVs and indels identified in the 15 genomes. **a** Heterozygosity proportion of SNVs (green) and indels (pink) identified from 15 individuals. The number of homozygous alternative variants and heterozygous variants for each population were calculated separately. The heterozygosity proportion was calculated as the number of heterozygous variants divided by the sum of the number of homozygous alternative variants and heterozygous variants. **b** Proportion of novel SNVs (green) and novel indels (pink) in all 15 individuals with the mean mapped depth. **c** Number of SNVs with different impacts per sample. (**d**) Number of indels with different impacts per sample

The five native Malaysian trios were sequenced at coverage of 28–38× (~ 30× on average; Additional file 1: Table S1). One Mendriq (MDQ) sample had the lowest sequence coverage at 28.3× (Table 1). On average 97.5% (Phred Score ≥ 10) of the reads were mapped to the reference genome (GRCh37). As shown in Table 1, more than 6.9 million SNVs (3.4 million per genome) and approximately 1.2 million bi-allelic indels (< 100 bp, 0.6 million per genome) were discovered in the fifteen genomes. The average Ti/Tv ratio was similar across all the native Malaysian populations (2.1 per genome), which was consistent with published reports [14, 38, 39]. The individual genome heterozygosity ranged between 51.6–56.7% for SNVs and 59.5–66.8% for indels, lower than other global populations (Table 1; Fig. 1a), suggesting that the native Malaysian populations are genetically more homogenous.

**Table 1:** Summary of sequence alignment and variants calling

	Bateq				Mendriq				Semai					Dusun				Murut	
	BTQ016	BTQ038	BTQ055		MDQ010	MDQ025	MDQ045		SMI018	SMI034	SMI041		NB07	NB08	NB09		NB10	NB11	NB12
*Sequencing depth*	37.71	36.34	37.55		35.72	28.32	35.38		36.03	36.26	36.55		35.79	37.44	37.56		36.96	37.73	36.52
*Bases covered percentage*																			
Phred score ≥ 5	98.34	98.3	97.78		97.68	97.44	98.12		98.29	97.79	98.32		98.3	97.79	98.35		98.35	97.8	98.31
Phred score ≥ 10	97.83	97.73	97.35		97.11	96.23	97.29		97.75	97.36	97.78		97.71	97.37	97.82		97.8	97.38	97.77
*Ti/Tv ratio*	2.105	2.114	2.106		2.105	2.096	2.105		2.107	2.107	2.115		2.1	2.098	2.101		2.105	2.096	2.103
*Novel variant proportion (%)*																			
SNPs	1.19	1.16	1.21		0.87	0.68	1.03		0.69	0.70	0.69		0.32	0.34	0.34		0.33	0.33	0.33
Indels	1.49	1.48	1.47		1.34	1.23	1.41		1.28	1.25	1.29		1.11	1.08	1.12		1.12	1.06	1.11
*Heterozygous variant proportion (%)*																			
SNPs	52.56	51.63	53.53		56.63	54.94	54.12		53.44	54.68	52.03		52.60	53.70	52.88		52.30	54.78	52.6
indels	59.96	59.53	60.91		66.85	65.24	64.79		60.44	62.04	59.67		60.50	61.42	61.75		60.18	62.12	60.14
*Number of variants in different types*																			
Synonymous SNPs	11,433	11,455	11,530		11,685	11,414	11,563		11,625	11,512	11,482		11,302	11,290	11,212		11,522	11,427	11,433
Non-symonymous SNPs	10,902	10,827	10,876		11,109	10,865	10,810		11,051	10,947	10,783		10,753	10,688	10,865		10,825	10,786	10,729
Stop-loss	31	27	28		26	21	29		24	24	23		26	31	34		30	29	27
Stop-gain	88	86	99		104	100	97		101	92	103		111	107	111		93	104	99
Small frameshift indels	345	332	346		317	291	312		337	329	332		311	310	323		327	352	344

We further examined whether there were genomic regions enriched with variants. Hotspots of variants were determined by selecting the top 1% non-overlapping windows across the genome, each spanning 1 Mb, with top counts of mutations that passed the quality control (genotyping quality ≧ 50; read depth = 10–120; allele balance = 0.3–0.7). SNVs and indels were treated independently. Regions adjacent to 1 Mb from the telomeres and centromeres were excluded. As expected, the region Chr6:29–33 Mb harboured the largest number of both SNVs and indels, followed by Chr8:3–4 Mb (Additional file 1: Table S2-S3). These two regions encompass immunity-related protein-coding genes (the MHC Class II genes, *ANGPT2*, *DEFA*, and *DEFB* on chromosome 6; *CSMD1* on chromosome 8) [40–42], and have been reported previously as SNV hotspots in the Singapore Malays [38]. Particularly noteworthy is *CSMD1,* which is highly expressed in the brain [43] and may play a role in the susceptibility of malarial infection [41, 42]. The region Chr22:49–50 Mb was another hotspot of SNVs and indels, spanning two immune related genes *FAM19A5* and *C22orf34*. Protein-coding genes underlying the mutation hotspots regions were significantly enriched in olfaction, immunity and hemoglobin among others (Additional file 2: Table S4), suggesting that genomic regions which are ‘sensitive’ towards environmental responses tend to be more variable.

We applied SnpEff version 4.3 T [36] to classify the variants according to their functional effects, and summarized the number of SNVs and indels of each category in each population in Additional file 1: Table S5-S6. We found 98.5% of the SNVs and 99% of the indels were non-coding variants; while the remains included possibly harmful variants with low (1.1% SNVs and 0.08% indels) and moderate (0.4% SNVs and 0.15% indels) impact, and disruptive variants with high impact (0.03% SNVs and 0.07% indels, e.g., exon-loss, frameshift, splice-acceptor, splice-donor, start-lost, stop-gained, stop-lost, and transcript-ablation variants). Each genome carried 290 loss-of-function (LOF) SNVs on average (Additional file 1: Table S7), consistent with previously reported number of LOF variants (200–800) in each healthy human genome [44]. Although fewer samples were sequenced, the number of LOF-SNVs in our data was comparable with that reported in the 1000 Genomes Project data (Additional file 1: Table S5), which represents a larger sample size with low sequencing depth. When comparing across the five native Malaysian populations, the number of LOF-SNVs per genome between OA and NB were similar (291 vs. 289 per sample) (Fig. 1c; Additional file 1: Table S7).

On average, 486 high-impact indels and 320 LOF-indels were identified in each sample, similar with other global populations (Fig. 1d; Additional file 1: Table S6-S7) [45]. Of these, 354 were homozygous deletions in at least one sample, and 555 indels presented in more than one sample. Frameshift indels (FS-indels) are generally thought to be pathogenic and may confer significant phenotypic consequences [45]. We observed 644 FS-indels in the 15 samples (on average 327 in each), of which 171 were homozygous deletion in at least one sample, and 580 FS-indels presented in more than one sample. One example of high-frequency FS-indels in the 15 samples is an 11-bp mutation affecting *MICA* (frequency = 0.87). *MICA* has been attributed to autoimmune diseases and viral infection [46, 47]. Details of the FS-indels identified are tabulated in Additional file 3: Table S8. Protein-coding genes affected by LOF-indels showed significant enrichment in Ca^2+^-dependent cell adhesion and olfactory transduction (Additional file 2: Table S4). A similar functional enrichment pattern was observed on genes overlapping with FS-indels.

### Identification of novel SNVs and indels

We observed approximately 0.19 million SNVs (2.7%) and 0.03 million indels (2.3%) not reported in dbSNP153. The overall novelty rate across autosomal chromosomes was similar, ranging from 2.2% (chromosome 21) to 3.0% (chromosome 5) for SNVs, and from 2.0% (chromosome 13) to 2.9% (chromosome 22) for indels. Genomic regions emerged with higher densities of novel SNVs or indels are listed in Additional file 1: Table S9-S10. The variant-enriched region Chr8:3–6 Mb, again, harbored the largest number of novel SNVs; Chr1:145–148 Mb showed a substantial excess of novel indels than other regions.

Comparing across the native Malaysian populations, we found that OA populations harbored more novel variants than NB populations did on both population (1.0–1.6% of SNVs and 1.4–1.7% of indels in OA; 0.5% of SNVs and 1.2% of indels in NB) and individual (0.7–1.2% of SNVs and 1.2–1.5% of indels in OA; 0.3% of SNVs and 1.1% of indels in NB) levels (Table 1; Fig. 1b; Additional file 1: Table S5-S6). Notably, the two Negrito populations especially the Bateq (BTQ) trio, harbored the highest proportion of putative novel SNVs and novel indels (novelty rates are 1.2% for SNVs and 1.5% for indels in each BTQ sample) (Additional file 1: Table S5-S6). OA and NB populations shared a smaller number of novel SNVs (1323, making up 0.9 and 3.1% of the novel SNVs in OA and NB, respectively), but more novel indels (8358, making up 36.4 and 64.7% of the novel SNVs in OA and NB, respectively) in common.

### Estimating de novo mutation rates

We further identified autosomal de novo mutations in the offspring of each trio. We applied stringent control for genotyping quality, and found that the sequencing depth and mapping quality at these de novo variants are not significantly lower than the genome-wide level, and most of them (94.5%) are located outside the simple repeats region (Additional file 1: Fig. S1). We also filtered out the mutations with allele balance ≤0.3 or ≥ 0.7. Therefore, the de novo mutations identified could be considered in the germline (see Methods). The number of de novo SNVs ranged in 37–62 for each offspring (listed in Additional file 1: Table S11). Correspondingly, the germline de novo mutation rate was estimated to be 0.81 × 10^− 8^-1.33 × 10^− 8^ per site per generation for SNVs (Table 2), which falls within the expected range [15, 48]. As listed in Additional file 1: Table S11, there were a total of 242 de novo SNVs in the five offsprings, affecting 137 genes, of which 108 were protein-coding genes. These genes showed significant functional enrichment in epidermal growth factor (8 of the 108 genes, Additional file 2: Table S4). All the de novo SNVs were individual-specific, but we found two mutations in MDQ (Chr2:141,474,240) and Dusun (DSN) (Chr2:141,657,309) falling in the same gene, *LRP1B*, which encodes for a member of the low-density lipoprotein receptor family. In addition, *CACNA1C* and *SLC43A2* were affected by multiple de novo SNVs in MRT. Two adjacent intronic allele substitutions (at positions 2,605,335 and 2,605,336, respectively; both were novel mutations) occurred in *CACNA1C*. This gene encodes a subunit of voltage-dependent calcium channel, and plays important roles in a wide range of biological functions, e.g. muscle contraction, hormone or neurotransmitter release, gene expression, cell motility, cell division and cell death, and might be attributed with cardiovascular diseases. Other interesting de novo SNVs include a ‘modifier’ C > T substitution at rs72668090 in *EGLN3* and a T > C mutation at position 84,692,399 in *NRG3* in the MDQ offspring. Both genes were reported to function in cardiovascular diseases [49, 50].

**Table 2:** Autosomal de novo mutation rates for SNV, indel and CNV in each trio

Population	SNV		Indel		CNV		
	# de novo mutations	Mutation rate (10^−8^)	# de novo mutations	Mutation rate (10^− 8^)	# de novo mutations	# total mutations	Mutation rate
Bateq	49	1.08	5	0.13	2	1754	0.001
Mendriq	37	0.81	10	0.29	1	2172	0.0005
Semai	40	0.86	6	0.15	3	1722	0.002
Dusun	54	1.2	4	0.1	4	1727	0.002
Murut	62	1.33	8	0.21	2	1777	0.001

The mutation rates (per site per generation) for SNV and indel were estimated using a callability-based approach (see Methods), and that for CNV was calculated as the number of de novo mutations divided by the total mutations

Compared with SNVs, the de novo mutation events for indels occurred less frequently. The mutation rate was estimated to be 1.0 × 10^− 9^-2.9 × 10^− 9^ per site per generation according to the 4–10 de novo indels identified in each offspring (Table 2; Additional file 1: Table S12), in accordance with previous reports [14, 52]. We did not observe any direct physical or functional attribution between the de novo indels and de novo SNVs in each sample – they were located distant from each other (> 1 Mb) and in different genes. A candidate gene of interest affected by a de novo indel was *CDH13* in the Murut (MRT) offspring. *CDH13* is a member of GPI-anchored member of the cadherin superfamily, which encodes for the protein T-cadherin that is prominently expressed in heart. It is associated with blood pressure regulation, atherosclerosis protection and regulation of adiponectin level [52, 53]. Interestingly, this gene was also reported to be associated with malaria susceptibility [54], and consistently exerted as a signature of positive selection in the Negrito populations from Peninsular Malaysia [17, 18].

### Analysis of copy number variants

**Fig. 2:**
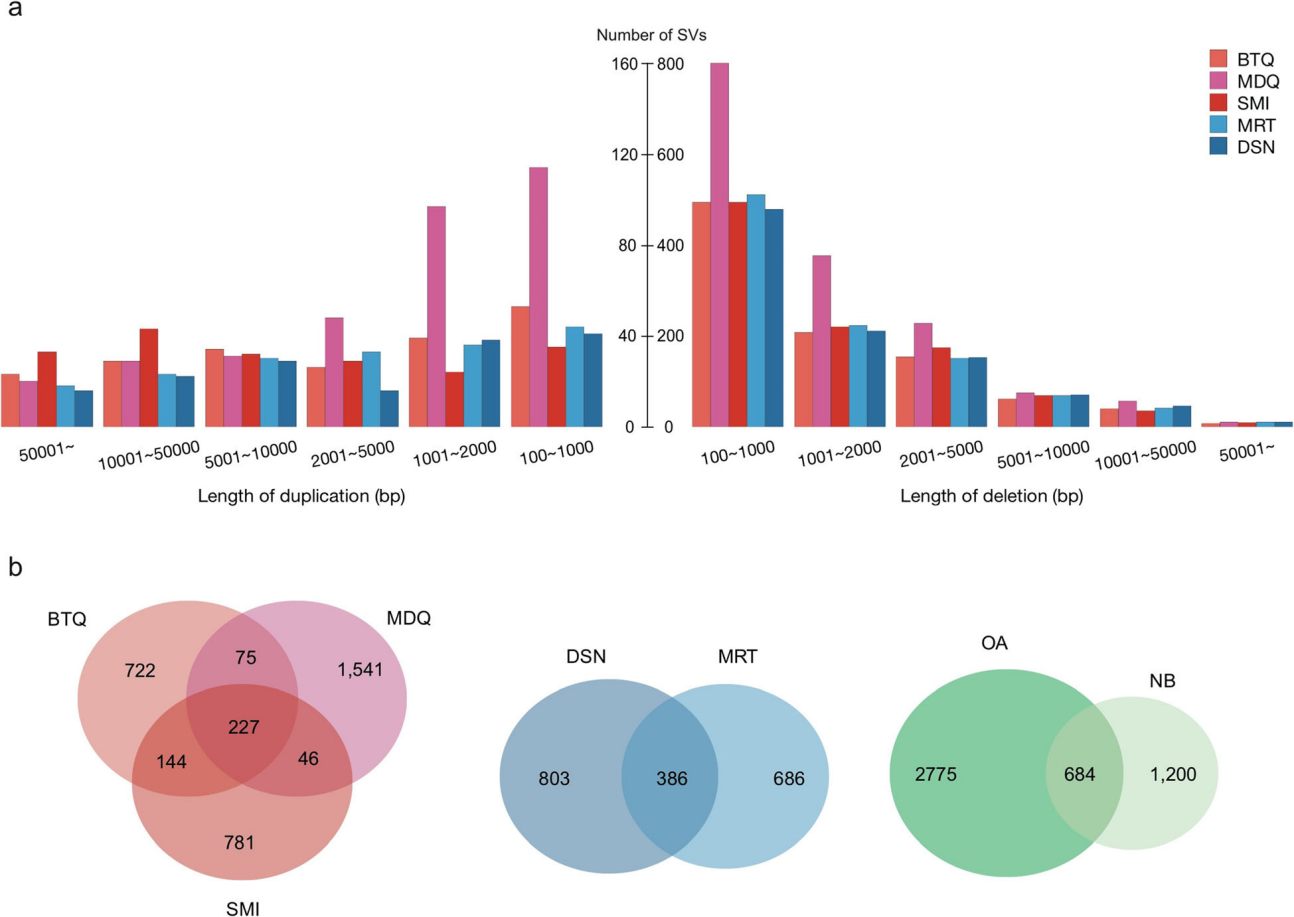
Structural variants identified in the 5 trios. **a** The number and length distribution of the duplications (left) and deletions (right) in each trio. **b** Venn diagrams represented the number of shared and unique SVs among three OA trios (left), within two NB trios (medium) and between these two groups (right)

To minimize potential false positive calls, we utilized both ERDS and CNVnator to identify CNVs on the individual level (see Methods). Consequently, 9152 CNVs over 100 bp in size were detected in the 15 samples, including 7470 deletions and 1682 duplications. Each sample carries 551–777 CNVs (610 on average) (Additional file 1: Table S13**)**. The number of CNVs identified in each genome was similar (~ 1700), except that the MDQ trio was observed to carry a higher number of CNVs (2172). The size distribution of CNVs is shown in Fig. 2a. Deletions were enriched in the length of 461 bp (43 deletions), and duplications were enriched in the length of 1 kb (458 duplications). The largest CNV was a duplication found in the MRT trio, spanning 529 kb at 18q11.2. It encompassed *RBBP8*, which encodes for protein that regulates cell cycles and proliferation [55]. Using the 50% reciprocal overlap criteria to compare with the Database for Genome Variants (DGV), a substantial amount of the CNVs identified (~ 22.1%; 742 deletions and 1276 duplications) are previously unreported, of which 1214 (13%) were recurrent (observed in at least 2 out of the 15 genomes studied). These novel CNVs were enriched in size range < 1 kb for deletions and in 1–10 kb for duplications. In the total of 9152 CNVs, 42% (3832) were genic variants, disrupting 694 genes (i.e, CNV breakpoints fell within the exons; average 139 genes per genome). We observed a large number of duplications (copy number (CN) > 2) in this study, which suggests that the duplication events may have been under-reported in previous array-based platforms, likely due to the limitation of the nature of the technology. We observed 1–4 de novo CNVs in each offspring, which converts to a mutation rate of ~ 0.001, consistent with the range of the reported rate (Table 2; Additional file 1: Table S14) [48]. All the 12 de novo CNVs were deletions ranging in 281–2778 bp. Two candidate genes of interest affected by the de novo CNVs were *LMF1* and *CLDN14* identified in MDQ and DSN, respectively. *LMF1* encodes for protein lipase maturation factor, which involves in maturation and transport of lipase. *CLDN14* encodes an integral membrane protein and a component for tight junction strands regulating the cell-cell adhesion in epithelial or endothelial cell sheets.

We then investigated the CNV sharing among the native Malaysian trios, and grouped them as Orang Asli CNVs (OA-CNVs; shared by BTQ, MDQ and Semai (SMI)), Negrito CNVs (NGO-CNVs; shared by BTQ and MDQ), North Bornean CNVs (NB-CNVs; shared by DSN and MRT), and Malaysian CNVs (MLS-CNVs; shared by OA and NB populations). As expected, populations that are historically closer tended to share more CNVs. For instance, we observed more CNV regions shared within OA population (302 OA-CNVs) and within NB population (386 NB-CNVs), than those shared between these two groups (227 MLS-CNVs) (Fig. 2b; Additional file 4: Table S15). Candidate genes affected by the OA-CNVs were enriched in the synapse-related ion transduction (Additional file 2: Table S4).

We further investigated the inheritance of several candidate genes of interest that were known to either lie on the segmental duplication region, or carry multiple allelic CNVs. Numerous studies have reported the attributions and roles of CNVs underlying these genes in a wide range of disease traits. Genes affected by those reported CNVs are listed in Additional file 1: Table S16, including: *CCL3L1*, *DEFA/B*, *FCGR2/3*, *AMY1/2*, *GSTT/GSTM*, *LPA*, and *CYP2D6* [56–65]. The copy number of these candidate genes were surprisingly lower than average as previously reported [57, 58, 60, 66]. All five trios showed duplication (copy number = 3) in *AMY* and *DEFB103A* (except MRT) but a deletion (copy number = 1) in gene *DEFB130*. BTQ and MDQ showed duplication for the *DEFA1B* gene (copy number > 2) but not the rest of the trios. The most variable gene among all trio members were *LPA*, ranging in 4 (DSN) -10 (MDQ). Some of the copy number of these candidate genes of interest were not called, probably due to the stringent quality control criteria during the SV call, which had filtered out the ‘noisy’ calls. Validation is recommended for identifying these copy number variants harbouring the complex and segmental duplicated regions [57, 67–69].

### Novel insertions shared with archaic hominids

Novel insertions (NIs) (fragment size in 47–19,045 bp) to the human reference genome have been identified in the native Malaysian genomes. To avoid possible artefacts in sequencing and variants calling, we only focused on the inherited NIs that was present in the offspring and at least one of his/her parent. The number of inherited autosomal NIs in OA (5984–6145 in each trio) was slightly more than that in NB (5979–5991 in each trio), probably due to the ancient origin and long-term isolation of the OA hunter-gatherers. A full list of inherited NIs can be found in Additional file 5: Table S17. We found 547 of these NIs were unique to the OA, and 205 were unique to NB. Interestingly, 45.2–46.4% of the NIs in each sample could also be observed in the Neanderthal or Denisovan genomes (Table 3). Consistent with the lower proportion of Denisovan-like genomic segments in the native Malaysian populations [23], only around 50 of the archaic-like NIs in each sample could be specifically observed in the Denisovan genome; while the Neanderthal-like segments constituted a large proportion of the archaic-like NIs. Enrichment analysis revealed that genes underlying Neanderthal-like segments are enriched for synapse-related cell junction, immunity, ion channel, etc. (Additional file 2: Table S4).

**Table 3:** Inherited novel insertions in the 5 native Malaysian trios

Population	# Total NIs	# Archaic-like NIs	# Neanderthal-like NIs	# Denisovan-like NIs
Bateq	5984	2765	1873	48
Mendriq	6145	2776	1889	49
Semai	6010	2790	1920	47
Dusun	5991	2741	1862	49
Murut	5979	2742	1859	52

Archaic-like NIs, NIs that could be found in the Neanderthal or Denisovan genomes; Neanderthal-like NIs, archaic-like NIs that could be specifically found to the Neanderthal genome; Denisovan-like NIs, archaic-like NIs that could be specifically found to the Denisovan genome

## Discussion

This study presents for the first time, a comprehensive catalogue of genomic variants of the native trio inhabitants from Peninsular Malaysia and North Borneo. Although whole-genome sequencing has been commonly applied in population genomic studies, very few publications have reported on ‘healthy’ trios [14, 15, 51], and of particular interest are the lack of data on deep-sequenced trios of native inhabitants such as the Orang Asli and North Borneans. Identification of variants and the frequencies of these populations could provide better insights to population-specific disease susceptibility and serve as an important stepping-stone for advancing clinical and public health genomic study [70]. Studying trios allows us to confirm rare and putatively population specific variants that are often of clinical importance but under-represented, since they are being transmitted, therefore of high confidence.

On a separate note, sequencing of trios remains the most straightforward strategy to estimate de novo mutation rate [15]. This is crucial to date the evolutionary events and to investigate the underlying causes for genetic diseases [10]. We caution that de novo mutations may be affected by paternal age [71, 72], which was not available in our data. However, the estimated rate of de novo mutations observed in this study is in line with other recent studies [15, 48], and is not likely to be affected by sequencing error or low mapping quality (Additional file 1: Figure S1, Table S11-S12). De novo mutations are often thought to undergo a different nature of selective pressure and are more deleterious than the inherited variants, thus, are more likely to be the causative factor for many diseases [27, 48]. The de novo SNVs in the genomes studied here (Table 2) were enriched in genes related to EGF related pathways. Earlier study have shown that EGF receptor pathway may show adaptive changes against micro-environmental forces specifically hypoxia, acidosis and reactive oxygen species, thus able to induce genetic instability [73].

Haplotype phasing on trios using identity by descent (IBD) essentially is thought to be more informative and accurate than the phasing of unrelated individuals based on the haplotype frequency information [74, 75]. When both parents are genotyped, variants that are not triply heterozygous in the parents and offspring could be phased. The IBD-based phasing using the trios of this study, along with the population haplotype frequency models, could be used as a reference and applied to additional populations, especially the Southeast Asians. It works particularly for imputing variants with low frequency. The task of phasing in isolated populations is somewhat a special case, as individuals from such populations exhibit much higher levels of relatedness, and tend to share much longer stretches of IBD-sequences than a pair of unrelated individuals from a non-isolated population.

Higher discovery rate of novel variants was observed in the trio genomes from Peninsular Malaysia and North Borneo compared to the global populations (Table 2) possibly implies an insufficient coverage of population diversity in the attempt of unveiling the genome architecture. Therefore, more sequencing attempts on the indigenous and more diversified populations (in particular the Southeast Asia region) should be carried out in the near future.

In line with the genetic relationship among the native Malaysian populations, more CNVs were shared among OA populations and among NB populations than across the two groups. Our functional enrichment analyses suggested that the genes underlying CNVs in OA differed from those in NB. The OA-specific CNVs were enriched in genes involved in immunity; whereas the NB-specific CNVs were enriched in protein secretory related pathways, suggesting possibility of different population history between these two different groups. In addition, we observed putatively NIs shared with the archaic hominid in the OA and NB populations, and they are significantly enriched in post-synapse membrane and Pleckstrin homology-like domain. Given the potential roles of the underlying candidate genes, collectively these enrichments imply that archaic hominin introgression may have helped to shape the specificity of the native Malaysian populations, and could possibly affect the regulation of immune response of these populations. Further investigations are warranted in order to provide further insights to the evolutionary process of immune systems of anatomical modern humans.

Several limitations were acknowledged in this study. First, recruitment of trios from the natives like OA and NB has been challenging because their population size is small, and identification of the biological matched trio members had been rather difficult. This limitation indeed hindered population-based analyses of this study. Second, there is a gap to precisely identify breakpoints of the CNVs called, due to the limitation of technology and power of the analysis tools.

## Conclusions

In this paper, we analyzed the deep-sequenced genomes of five native trios from Peninsular Malaysia and North Borneo, and presents a full catalogue of the genome variants. It has several important implications to regional human population genetics. First, the higher discovery rate of novel variants in our genomes, although with a small sample size, compared to global populations suggested insufficient coverage of population diversity in the existing map of genomic variations, hence emphasizing the necessity of conducting further genomic studies on ‘minor’ populations of the world, such as the native inhabitants from Malaysia. Second, the estimated mutation rate and accurate phasing of the trio haplotypes could potentially be used as a reference to genomic studies of similar populations. Third, the differentiation of OA and NB genomes imply discrepant demographic history of these two populations, in accordance with previous studies [20]. Last, the inherited novel insertions with shared with archaic hominids identified in our samples may imply unique population history of the native inhabitants in Malaysia.

## Methods

### Sample collection and genome sequencing

Fifteen peripheral blood samples consisting of five sets of trios (father-mother-offspring) were collected from Malaysia under the approval of the Research and Ethics Committee of Universiti Teknologi MARA [Ref no: 600-RMI (5/1/6)], the Department of Orang Asli Development (Jabatan Kemajuan Orang Asli Malaysia, JAKOA) [JHEOA.PP.30.052.Jld 5(17)], and the Universiti Malaysia Sabah Medical Research Ethics Committee [code: JKEtika 4/10(3)], as well as the district offices, village chief, and the chairperson of the Committee of Village Development and Security. Informed written consent was obtained from the volunteers aged 18 years and above. Their family history, pedigree, and self-reported ethnicity were recorded via an interview using local dialect. These five trios were from five native groups (one for each). Three of them were from Peninsular Malaysia, including Bateq (BTQ), Mendriq (MDQ) (both are the Negrito tribes) and the Semai (SMI) (Senoi) groups. They are collectively known as Orang Asli (OA). The other two were from North Borneo (abbreviated as NB population), including Dusun (DSN) and Murut (MRT) populations. The information of the trios is listed in Additional file 1: Table S1. The sampling procedure, as well as the protocol for genome sequencing were described in detail in Yew et al. (2018) [23]. Briefly, genomic DNA was isolated from peripheral samples using DNeasy Blood and Tissue kit (Qiagen, Hilden, Germany), and the integrity met the whole genome sequencing requirement (OD 260/280 reading ranging from 1.8–2.2). These samples were sequenced using Illumina HiSeq 2000, with a mean coverage of 30× (range 28–38×), targeted for 100 bp paired-end reads, with insert sizes of 300–400 bp according to the manufacturer’s instruction.

### Read mapping and variant calling

Briefly, the pair-end reads in fastQ files were mapped to human reference genome GRCh37 with BWA 0.7.5a [24], and were subsequently merged and sorted to BAM format using samtools 0.1.16 [25]. Low quality reads and potential duplicates produced by the polymerase chain reaction in the library construction were removed. We then realigned the reads mapped around potential small insertion or deletions using GATK 2.2–3 [26], and recalibrated the base quality scores. Reads with mapping quality (MQ) larger than 30 were kept for trio-aware variants calling with unified genotyper module in GATK2. Details of variants calling can be found in Yew et al. (2018) [23].

### Identification of de novo SNVs and indels

A variant in the offspring was defined as a de novo one if it presents in neither parent but in the offspring of a trio. In detail, it should meet the following criteria: i) the locus did not fall in the regions with poor mappability, low complexity or with enriched aberrant SNPs (as reported by Mallick et al.) [2]; ii) the read depth at this locus should be between 10 and 120, and 30–70% of the reads should support the alternative allele (allele balance in 0.3–0.7), as suggested by Kong et al. [27] and Neale et al. [28], to remove putative CNV regions in the offspring where the reads from highly similar regions are often mixed together; iii) the genotype quality of the variant should be ≧ 50 in both offspring and parents; iv) no read supports the alternative allele in each parent; and v) the alternative allele should be absent from both parents but present in the offspring.

### Identification of copy number variants (CNVs)

We defined autosomal CNVs in this study as deleted or duplicated DNA segments larger than 100 bp to distinguish it from the indels (< 100 bp). CNVs were identified using CNVnator [29] and ERDS [30] in combination as recommended by Trost el al. (2018) [31]. A filtered set of CNVs was then generated by removing those variants that were: i) identified by only one method (< 50% overlap); ii) overlapped with any repetitive and low-complexity regions; or iii) with ≥50% overlap with gaps and segmental duplications.

### Identification of novel and de novo CNVs

A novel CNV was defined if it had < 50% overlap with a CNV region reported in the Database for Genome Variants (DGV) (http://dgv.tcag.ca/dgv/app/home, GRCh37 [32]). We defined a de novo CNV as a deletion or duplication that was only present in the offspring but absent in both parents. The following steps of stringent criteria were used to identify de novo CNVs: (i) we compared whether the putative CNV in the offspring was present in his/her parents with consistent type (i.e copy number gain or copy number loss) under the reciprocal overlapping threshold of 50%; (ii) CNV that was not found in step (i) were genotyped by CNVnator in the trios, and we then filtered out the variants of which the genotyping results were consistent in the trios; (iii) we subsequently manually checked all the remaining CNVs in the last step to confirm if the variant was indeed true de novo.

### Estimating the de novo mutation rate

We calculated de novo mutation rate for SNVs and indels in each trio based on a callability-based method in which the probability of each site that can be called as a de novo mutation is considered in denominator rather than simple counts of sites [15]. For a site *s* with an actual de novo mutation, the callability *C*_*k*_(*s*) is defined by the probability of calling *s* as a de novo mutation in the family k. The de novo mutation rate of a family *k* is

$$ {\mu}_k=\frac{\left| de\  novo\ \mathrm{mutaions}\ \mathrm{in}\ \mathrm{family}\ k\right|}{2\times {\sum}_{s\in sites}{C}_k(s)} $$

For CNVs, the de novo mutation rate was calculated as the number of de novo CNVs divided by the total CNVs in the offspring and averaged across trios.

### Analysis of novel insertions

We used PopIns [33] with default settings to assemble the human reference genome (GRCh37) unmapped reads from the five trios into contigs together with those unmapped reads from the archaic hominin genome sequences (e.g., Neanderthal and Denisovan, obtained from http://cdna.eva.mpg.de/neandertal/altai/) [34, 35]. These assembled contigs were candidate novel sequences which were absent from the human reference genome. We further filtered out the novel sequences in the trios if they violated Mendelian inheritance as they are more likely to be false positives.

### Variants annotation and functional enrichment analysis

Annotation of the variants (SNVs, indels and CNVs) were performed using SnpEff version 4.3 T [36], which provide estimated biological effects for each variant. Functional enrichment analysis of each set of gene was conducted using the database for annotation, visualization and integrated discovery (DAVID) v6.8 [37]. Enrichment score > 1.3, as proposed by the authors [37], and Benjamini-FDR-corrected *p* value < 0.05 were considered as thresholds of significance.

### Haplotype phasing

Parent-trio haplotype phasing was carried out using SHAPEIT for the five trios [5]. SHAPEIT allows for the inference of haplotypes using identity-by-descent (IBD) at any sized pedigrees. Prior to the haplotype phasing, we removed 39,429 SNPs, 31,657 indels, and 77 CNVs with missing rate > 10% in the fifteen samples or exhibiting Mendelian error in two or more trios. For each trio, SNVs, indels and CNVs were phased in a combined dataset.

## Supplementary information

**Additional file 1: Figure S1.** Data quality of the de novo variants. **Table S1** Sample information. **Table S2.** Summary information of genomic regions with top 1% of SNV density over the genome. **Table S3.** Summary information of genomic regions with top 1% of indel density over the genome. **Table S5.** Functional annotation of SNVs in each native population and global populations. **Table S6.** Functional annotation of indels in each native population and global populations. **Table S7.** Functional annotation of SNVs and indels in each native Malaysian genome. **Table S9.** Genomic regions identified as novel SNV hotspots. **Table S10.** Genomic regions identified as novel indel hotspots. **Table S11.** List of the de novo SNVs identified in each offspring. **Table S12.** List of the de novo indels identified in each offspring. **Table S13.** Summary of CNVs identified in each trio. **Table S14.** De novo CNVs identified in the 5 off-springs. **Table S16.** Inheritance of selected genes that are known to either lie on the segmental duplication regions, or carry CNVs.

**Additional file 2: Table S4.** Functional enrichment of genes underlying the mutation hotspots, loss-of-function variants, de novo variants, copy number variants and novel insertions.

**Additional file 3: Table S8.** Distribution of FS indels and the candidate genes affected.

**Additional file 4: Table S15.** CNVs sharing across the native Malaysian trios.

**Additional file 5: Table S17.** Inherited novel insertions in the 5 native Malaysian trios.

## Data Availability

The genome sequences of the samples are available in the National Omics Data Encyclopedia (NODE) (http://www.biosino.org) with the assigned accession number: NODEP00371760. SVs detected in the samples have been deposited to dbVar (https://www.ncbi.nlm.nih.gov/dbvar/) with the accession number: nstd172.

## Abbreviations

BTQ: Bateq
CNV: Copy number variant
DAVID: Database for Annotation, Visualization and Integrated Discovery
DGV: Database for Genome Variants
DSN: Dusun
FS: Frameshift
IBD: Identity by descent
indel: Insertions and deletions
LOF: Loss-of-funtion
MDQ: Mendriq
MQ: Mapping quality
MRT: Murut
NB: North Bornean
NI: Novel insertion
OA: Orang Asli
SMI: Semai
SNV: Single nucleotide variant
SV: Structural variant

## References

[1] The 1000 Genomes Project Consortium (2015). A global reference for human genetic variation. Nature.

[2] Mallick S, Li H, Lipson M, Mathieson I, Gymrek M, Racimo F (2016). The Simons genome diversity project: 300 genomes from 142 diverse populations. Nature.

[3] Pagani L, Lawson DJ, Jagoda E, Mörseburg A, Eriksson A, Mitt M (2016). Genomic analyses inform on migration events during the peopling of Eurasia. Nature.

[4] Walter K, Min JL, Huang J, Crooks L, Memari Y, McCarthy S (2015). The UK10K project identifies rare variants in health and disease. Nature.

[5] Delaneau O, Marchini J, Zagury J (2011). A linear complexity phasing method for thousands of genomes. Nat Methods.

[6] Sawyer S, Hartl DL (1992). Population genetics of polymorphism and divergence. Genetics..

[7] Felsenstein J, Churchill GA (1996). A Hidden Markov Model Approach Evolution. Mol Biol Evol.

[8] Siepel A, Bejerano G, Pedersen JS, Hinrichs AS, Hou M, Rosenbloom K (2005). Evolutionarily conserved elements in vertebrate, insect, worm, and yeast genomes. Genome Res.

[9] Cooper GM, Stone EA, Asimenos G, Green ED, Batzoglou S, Sidow A (2005). Distribution and intensity of constraint in mammalian genomic sequence. Genome Res.

[10] Veltman Joris A., Brunner Han G. (2012). De novo mutations in human genetic disease. Nature Reviews Genetics.

[11] Jin Z-B, Wu J, Huang X-F, Feng C-Y, Cai X-B, Mao J-Y (2017). Trio-based exome sequencing arrests de novo mutations in early-onset high myopia. Proc Natl Acad Sci.

[12] Yuen RKC, Merico D, Cao H, Pellecchia G, Alipanahi B, Thiruvahindrapuram B (2016). Genome-wide characteristics of de novo mutations in autism. NPJ Genomic Med.

[13] Al-Mubarak B, Abouelhoda M, Omar A, Aldhalaan H, Aldosari M, Nester M (2017). Whole exome sequencing reveals inherited and de novo variants in autism spectrum disorder: a trio study from Saudi families. Sci Rep.

[14] Hai DT, Thanh ND, Trang PTM, Quang LS, Hang PTT, Cuong DC (2015). Whole genome analysis of a Vietnamese trio. J Biosci.

[15] Besenbacher S, Liu S, Izarzugaza JM, Grove J, Belling K, Bork-jensen J (2015). Novel variation and de novo mutation rates in population-wide de novo assembled Danish trios. Nat Commun.

[16] Combrink HJB, Soderberg C, Boutin ME, Boutin AY, Wise MR, Zook M (2008). Indigenous groups of Sabah: an annotated bibliography of linguistic and anthropological sources. 2nd editio.

[17] Deng L, Hoh BP, Lu D, Fu R, Phipps ME, Li S (2014). The population genomic landscape of human genetic structure, admixture history and local adaptation in peninsular Malaysia. Hum Genet.

[18] Liu X, Yunus Y, Lu D, Aghakhanian F, Saw WY, Deng L (2015). Differential positive selection of malaria resistance genes in three indigenous populations of peninsular Malaysia. Hum Genet.

[19] Deng L, Hoh B-P, Lu D, Saw W-Y, Twee-Hee Ong R, Kasturiratne A (2015). Dissecting the genetic structure and admixture of four geographical Malay populations. Sci Rep.

[20] Yew CW, Minsong A, Tiek S, Lau Y, Pugh-kitingan J, Ransangan J (2018). Genetic relatedness of indigenous ethnic groups in northern Borneo to neighboring populations from Southeast Asia , as inferred from genome-wide SNP data. Ann Hum Genet.

[21] The HUGO Pan-Asian SNP Consortium (2009). Mapping human genetic diversity in Asia. Science.

[22] Fu R, Mokhtar SS, Phipps ME, Hoh B-P, Xu S, Shuhada S (2018). A genome-wide characterization of copy number variations in native populations of peninsular Malaysia. Eur J Hum Genet.

[23] Yew C, Lu D, Wong L, Twee-Hee Ong R, Lu Y, Wang X (2018). Genomic structure of the native inhabitants of peninsular Malaysia and North Borneo suggests complex human population history in Southeast Asia. Hum Genet.

[24] Schneider VA, Graves-Lindsay T, Howe K, Bouk N, Chen HC, Kitts PA (2017). Evaluation of GRCh38 and de novo haploid genome assemblies demonstrates the enduring quality of the reference assembly. Genome Res.

[25] Li H, Handsaker B, Wysoker A, Fennell T, Ruan J, Homer N (2009). The sequence alignment/map format and SAMtools. Bioinformatics.

[26] McKenna A, Hanna M, Banks E, Sivachenko A, Cibulskis K, Kernytsky A (2010). The genome analysis toolkit: a MapReduce framework for analyzing next-generation DNA sequencing data. Genome Res.

[27] Kong A, Frigge ML, Masson G, Besenbacher S, Sulem P, Magnusson G (2012). Rate of de novo mutations and the importance of father-s age to disease risk. Nature.

[28] Neale B, Kou Y, Liu L, Ma’ayan A, Samocha K, Sabo A (2012). Patterns and rates of exonic de novo mutations in autism spectrum disorders. Nature.

[29] Abyzov A, Urban AE, Snyder M, Gerstein M (2011). CNVnator: an approach to discover, genotype, and characterize typical and atypical CNVs from family and population genome sequencing. Genome Res.

[30] Zhu M, Need AC, Han Y, Ge D, Maia JM, Zhu Q (2012). Using ERDS to infer copy-number variants in high-coverage genomes. Am J Hum Genet.

[31] Trost B, Walker S, Wang Z, Thiruvahindrapuram B, MacDonald JR, Sung WWL (2018). A comprehensive workflow for read depth-based identification of copy-number variation from whole-genome sequence data. Am J Hum Genet.

[32] MacDonald JR, Ziman R, Yuen RKC, Feuk L, Scherer SW (2014). The database of genomic variants: a curated collection of structural variation in the human genome. Nucleic Acids Res.

[33] Kehr B, Melsted P, Halldórsson BV (2016). PopIns: population-scale detection of novel sequence insertions. Bioinformatics.

[34] Prüfer K, Racimo F, Patterson N, Jay F, Sankararaman S, Sawyer S (2014). The complete genome sequence of a Neanderthal from the Altai Mountains. Nature.

[35] Meyer M, Kircher M, Gansauge M-T, Li H, Racimo F, Mallick S (2012). A high-coverage genome sequence from an archaic Denisovan individual. Science.

[36] Cingolani P, Platts A, Wang LL, Coon M, Nguyen T, Wang L (2012). A program for annotating and predicting the effects of single nucleotide polymorphisms, SnpEff. Fly (Austin).

[37] Huang DW (2009). Lempicki R a, Sherman BT. Systematic and integrative analysis of large gene lists using DAVID bioinformatics resources. Nat. Protoc.

[38] Wong L-P, Ong RT-H, Poh W-T, Liu X, Chen P, Li R (2013). Deep whole-genome sequencing of 100 southeast Asian Malays. Am J Hum Genet The American Society of Human Genetics.

[39] Wong LP, Lai JKH, Saw WY, Ong RTH, Cheng AY, Pillai NE (2014). Insights into the genetic structure and diversity of 38 south Asian Indians from deep whole-genome sequencing. PLoS Genet.

[40] Silver KL, Zhong K, Leke RGF, Taylor DW, Kain KC (2010). Dysregulation of angiopoietins is associated with placental malaria and low birth weight. PLoS One.

[41] Pozzoli U, Fumagalli M, Cagliani R, Comi GP, Bresolin N, Clerici M (2010). The role of protozoa-driven selection in shaping human genetic variability. Trends Genet.

[42] Ravenhall M, Campino S, Sepúlveda N, Manjurano A, Nadjm B, Mtove G (2018). Novel genetic polymorphisms associated with severe malaria and under selective pressure in North-Eastern Tanzania. PLoS Genet.

[43] Athanasiu L, Giddaluru S, Fernandes C, Christoforou A, Reinvang I, Lundervold AJ (2017). A genetic association study of CSMD1 and CSMD2 with cognitive function. Brain Behav Immun.

[44] Pelak K, Shianna KV, Ge D, Maia JM, Zhu M, Smith JP (2010). The characterization of twenty sequenced human genomes. PLoS Genet.

[45] MacArthur DG, Balasubramanian S, Frankish A, Huang N, Morris J, Walter K (2012). A systematic survey of loss-of-function variants in human protein-coding genes. Science.

[46] Garcîa G, Pêrez AB, Sierra B, Aguirre E, Kikuchi M, Sânchez L (2011). Association of MICA and MICB alleles with symptomatic dengue infection. Hum Immunol.

[47] Gambelunghe G, Gerli R, Bocci EB, Del Sindaco P, Ghaderi M, Sanjeevi CB (2005). Contribution of MHC class I chain-related a (MICA) gene polymorphism to genetic susceptibility for systemic lupus erythematosus. Rheumatology.

[48] Acuna-Hidalgo R, Veltman JA, Hoischen A (2016). New insights into the generation and role of de novo mutations in health and disease. Genome Biol.

[49] Lin Q, Huang Y, Booth CJ, Haase VH, Johnson RS, Celeste Simon M (2013). Activation of hypoxia-inducible factor-2 in adipocytes results in pathological cardiac hypertrophy. J Am Heart Assoc.

[50] Parsa A, Chang YPC, Kelly RJ, Corretti MC, Ryan KA, Robinson SW (2011). Hypertrophy-associated polymorphisms ascertained in a founder cohort applied to heart failure risk and mortality. Clin Transl Sci.

[51] Maretty L, Jensen JM, Petersen B, Sibbesen JA, Liu S, Villesen P (2017). Sequencing and de novo assembly of 150 genomes from Denmark as a population reference. Nature.

[52] Org E, Eyheramendy S, Juhanson P, Gieger C, Lichtner P, Klopp N (2009). Genome-wide scan identifies CDH13 as a novel susceptibility locus contributing to blood pressure determination in two European populations. Hum Mol Genet.

[53] Takeuchi T, Adachi Y, Ohtsuki Y, Furihata M (2007). Adiponectin receptors, with special focus on the role of the third receptor, T-cadherin, in vascular disease. Med Mol Morphol.

[54] Dastani Z, Hivert MF, Timpson N, Perry JRB, Yuan X, Scott RA, et al. Novel loci for adiponectin levels and their influence on type 2 diabetes and metabolic traits: a multi-ethnic meta-analysis of 45,891 individuals. PLoS Genet. 2012;8. 10.1371/journal.pgen.1002607 PMC3315470 22479202

[55] Band G, Le QS, Jostins L, Pirinen M, Kivinen K, Jallow M (2013). Imputation-based meta-analysis of severe malaria in three African populations. PLoS Genet.

[56] Grant GD, Brooks L, Zhang X, Mahoney JM, Martyanov V, Wood TA (2013). Identification of cell cycle – regulated genes periodically expressed in U2OS cells and their regulation by FOXM1 and E2F transcription factors. Mol Biol Cell.

[57] Hollox EJ, Hoh B-P (2014). Human gene copy number variation and infectious disease. Hum Genet.

[58] Walker S, Janyakhantikul S, Armour JAL (2009). Multiplex Paralogue ratio tests for accurate measurement of multiallelic CNVs. Genomics.

[59] Hollox EJ, Armour JAL, Barber JCK (2003). Extensive Normal copy number variation of a β-Defensin antimicrobial-gene cluster. Am J Hum Genet.

[60] MacHado LR, Hardwick RJ, Bowdrey J, Bogle H, Knowles TJ, Sironi M (2012). Evolutionary history of copy-number-variable locus for the low-affinity Fcγ receptor: mutation rate, autoimmune disease, and the legacy of helminth infection. Am J Hum Genet.

[61] Perry GH, Dominy NJ, Claw KG, Lee AS, Fiegler H, Redon R (2007). Diet and the evolution of human amylase gene copy number variation. Nat Genet.

[62] Zheng X, Feingold E, Ryckman KK, Shaffer JR, Boyd HA, Feenstra B (2013). Association of maternal CNVs in GSTT1/GSTT2 with smoking, preterm delivery, and low birth weight. Front Genet.

[63] Emeville E, Broquère C, Brureau L, Ferdinand S, Blanchet P, Multigner L (2014). Copy number variation of GSTT1 and GSTM1 and the risk of prostate cancer in a Caribbean population of African descent. PLoS One.

[64] Noureen A, Fresser F, Utermann G, Schmidt K (2015). Sequence variation within the KIV-2 copy number polymorphism of the human LPA gene in African, Asian, and European populations. PLoS One.

[65] Wu Z, Sheng H, Chen Y, Tang J, Liu Y, Chen Q (2014). Copy number variation of the lipoprotein ( a ) ( LPA ) gene is associated with coronary artery disease in a southern Han Chinese population. Int J Clin Exp Med.

[66] Beoris M, Amos Wilson J, Garces JA, Lukowiak AA (2016). CYP2D6 copy number distribution in the US population. Pharmacogenet Genomics.

[67] Hollox EJ, Huffmeier U, Zeeuwen PLJM, Palla R, Lascorz J, Rodijk-Olthuis D (2008). Psoriasis is associated with increased β-defensin genomic copy number. Nat Genet.

[68] Haridan US, Mokhtar U, Machado LR, Aziz ATA, Shueb RH, Zaid M (2015). A comparison of assays for accurate copy number measurement of the low-affinity FC gamma receptor genes FCGR3A and FCGR3B. PLoS One.

[69] Breunis WB, van Mirre E, Geissler J, Laddach N, Wolbink G, Van Schoot E Der, et al. Copy number variation at the FCGR locus includes FCGR3A, FCGR2C and FCGR3B but not FCGR2A and FCGR2B. Hum Mutat 2009;30:E640–E650. 10.1002/humu.20997 19309690

[70] Vendelbosch S, de Boer M, Gouw RATW, Ho CKY, Geissler J, Swelsen WTN (2013). Extensive variation in gene copy number at the killer immunoglobulin-like receptor locus in humans. PLoS One.

[71] Bustamante CD, Burchard EG, De la Vega FM (2011). Genomics for the world. Nature..

[72] Ségurel L, Wyman MJ, Przeworski M. Determinants of mutation rate variation in the human Germline. Annu Rev Genomics Hum Genet. 2014:1–24. 10.1146/annurev-genom-031714-125740 25000986

[73] Mills MB, Hudgins L, Balise RR, Abramson DH, Kleinerman RA (2012). Mutation risk associated with paternal and maternal age in a cohort of retinoblastoma survivors. Hum Genet.

[74] Gillies RJ, Verduzco D, Gatenby RA (2012). Evolutionary dynamics of carcinogenesis and why targeted therapy does not work. Nat Rev Cancer.

[75] Browning SR, Browning BL (2011). Haplotype phasing: existing methods and new developments. Nat. Rev. Genet..

[76] Zhang F, Deng HW (2010). Confounding from cryptic relatedness in haplotype-based association studies. Genetica.

