# Supplementary material for: Analysis of five deep-sequenced trio-genomes of the Peninsular Malaysia Orang Asli and North Borneo populations
Source: BMC Genomics. 2019 Nov 12;20:842. doi: 10.1186/s12864-019-6226-8 (PMC6852992; doi:10.1186/s12864-019-6226-8)
Supplement: Supplementary file 1 — Additional file 1: Figure S1. Data quality of the de novo variants. Table S1 Sample information. Table S2. Summary information of genomic regions with top 1% of SNV density over the genome. Table S3. Summary information of genomic regions with top 1% of indel density over the genome. Table S5. Functional annotation of SNVs in each native population and global populations. Table S6. Functional annotation of indels in each native population and global populations. Table S7. Functional annotation of SNVs and indels in each native Malaysian genome. Table S9. Genomic regions identified as novel SNV hotspots. Table S10. Genomic regions identified as novel indel hotspots. Table S11. List of the de novo SNVs identified in each offspring. Table S12. List of the de novo indels identified in each offspring. Table S13. Summary of CNVs identified in each trio. Table S14. De novo CNVs identified in the 5 off-springs. Table S16. Inheritance of selected genes that are known to either lie on the segmental duplication regions, or carry CNVs. [file 12864_2019_6226_MOESM1_ESM.docx]

**Additional figures and tables**

**Figure S1** Data quality of the *de novo* variants

(a) Read depth (DP) of the *de novo* SNPs and indels in the 15 genomes; (b) Read depth (DP) of the *de novo* SNPs and indels in the offspring genomes of the 5 trios; (c) Read mapping quality (MQ) of the *de novo* SNPs and indels. In each plot, the *p* value was obtained by two-sided Wilcoxon signed-rank test.

**Table S1** Sample information

| Population | Sample ID | Relationship | Location |
| --- | --- | --- | --- |
| Bateq | BTQ016 | Father | Peninsular Malaysia |
|  | BTQ055 | Mother | Peninsular Malaysia |
|  | BTQ038 | Offspring | Peninsular Malaysia |
| Mendriq | MDQ045 | Father | Peninsular Malaysia |
|  | MDQ025 | Mother | Peninsular Malaysia |
|  | MDQ010 | Offspring | Peninsular Malaysia |
| Semai | SMI018 | father | Peninsular Malaysia |
|  | SMI034 | mother | Peninsular Malaysia |
|  | SMI041 | offspring | Peninsular Malaysia |
| Murut | NB10 | father | North Borneo |
|  | NB11 | mother | North Borneo |
|  | NB12 | offspring | North Borneo |
| Dusun | NB07 | father | North Borneo |
|  | NB08 | mother | North Borneo |
|  | NB09 | offspring | North Borneo |

**Table S2** Summary information of genomic regions with top 1% of SNV density over the genome.

| Chrom-  osome | Start (Mb) | End (Mb) | # SNVs per Mb | Protein-coding Genes |
| --- | --- | --- | --- | --- |
| 8 | 3 | 7 | 6794.0 | *CSMD1, MCPH1, ANGPT2, AGPAT5, DEFB1, DEFA6, DEFA4, DEFA1, DEFA5* |
| 6 | 29 | 34 | 6223.2 | *OR2W1, OR2B3, OR2J1, OR2J3, OR2J2, OR14J1, OR5V1, OR12D3, OR12D2, OR11A1, OR10C1, OR2H1, UBD, GABBR1, OR2H2, MOG, ZFP57, HLA-F, HLA-G, HLA-A, ZNRD1, PPP1R11, RNF39, TRIM31, TRIM40, TRIM10, TRIM15, TRIM26, TRIM39, TRIM39-RPP21, RPP21, HLA-E, GNL1, PRR3, ABCF1, PPP1R10, MRPS18B, ATAT1, C6orf136, DHX16, PPP1R18, NRM, MDC1, TUBB, FLOT1, IER3, DDR1, GTF2H4, VARS2, SFTA2, DPCR1, MUC21, MUC22, C6orf15, PSORS1C1, CDSN, PSORS1C2, CCHCR1, TCF19, POU5F1, HCG27, HLA-C, HLA-B, MICA, MICB, MCCD1, DDX39B, ATP6V1G2-DDX39B, ATP6V1G2, NFKBIL1, LTA, TNF, LST1, NCR3, AIF1, PRRC2A, BAG6, APOM, C6orf47, GPANK1, CSNK2B, CSNK2B-LY6G5B-1181, LY6G5B, LY6G5C, ABHD16A, XXbac-BPG32J3.20, LY6G6F, MEGT1, LY6G6E, LY6G6D, C6orf25, LY6G6C, DDAH2, CLIC1, MSH5, MSH5-SAPCD1, SAPCD1, VWA7, VARS, LSM2, HSPA1L, HSPA1A, HSPA1B, C6orf48, NEU1, SLC44A4, EHMT2, C2, ZBTB12, CFB, NELFE, SKIV2L, DXO, STK19, C4A, AL645922.1, C4B, CYP21A2, TNXB, ATF6B, FKBPL, PRRT1, PPT2, PPT2-EGFL8, EGFL8, AGPAT1, RNF5, AGER, PBX2, GPSM3, NOTCH4, C6orf10, BTNL2, HLA-DRA, HLA-DRB5, HLA-DRB1, HLA-DQA1, HLA-DQB1, HLA-DQA2, HLA-DQB2, HLA-DOB, TAP2, PSMB8, PSMB9, TAP1, HLA-DMB, XXbac-BPG181M17.5, HLA-DMA, BRD2, HLA-DOA, HLA-DPA1, HLA-DPB1, COL11A2, RXRB, SLC39A7, HSD17B8, RING1, VPS52, RPS18, B3GALT4, WDR46, PFDN6, RGL2, TAPBP, ZBTB22, DAXX, KIFC1, PHF1, CUTA, SYNGAP1, ZBTB9, BAK1, GGNBP1, ITPR3, UQCC2, SBP1, IP6K3, LEMD2, MLN, GRM4* |
| 16 | 78 | 79 | 5399.0 | *VAT1L, CLEC3A, WWOX* |
| 8 | 13 | 16 | 5059.0 | *DLC1, C8orf48, SGCZ, TUSC3, MSR1* |
| 22 | 49 | 50 | 5054.0 | *FAM19A5, C22orf34* |
| 16 | 83 | 85 | 5052.0 | *CDH13, HSBP1, MLYCD, RP11-505K9.4, OSGIN1, NECAB2, SLC38A8, MBTPS1, HSDL1, DNAAF1, TAF1C, ADAD2, KCNG4, WFDC1, ATP2C2, TLDC1, COTL1, KLHL36, USP10, CRISPLD2* |
| 4 | 189 | 190 | 4934.0 | *TRIML2, TRIML1* |
| 8 | 17 | 19 | 4880.0 | *ZDHHC2, CNOT7, VPS37A, MTMR7, SLC7A2, PDGFRL, MTUS1, FGL1, PCM1, ASAH1, NAT1, NAT2, PSD3* |
| 16 | 5 | 9 | 4712.5 | *PPL, SEC14L5, NAGPA, ALG1, C16orf89, FAM86A, RBFOX1, TMEM114, METTL22, ABAT, TMEM186, PMM2, CARHSP1, USP7* |
| 5 | 2 | 3 | 4519.0 | *IRX2, C5orf38* |
| 8 | 1 | 2 | 4475.0 | *DLGAP2, CLN8, ARHGEF10, KBTBD11, MYOM2* |
| 11 | 5 | 6 | 4366.0 | *MMP26, OR51L1, OR52J3, OR52E2, OR52A5, OR52A1, OR51V1, HBB, HBD, HBG1, HBG2, HBE1, OR51B4, OR51B2, OR51B5, OR51B6, OR51M1, OR51J1, OR51Q1, OR51I1, OR51I2, OR52D1, UBQLN3, UBQLNL, OR52H1, OR52B6, TRIM6, TRIM6-TRIM34, TRIM34, TRIM5, TRIM22, OR56B1, OR52N4, OR52N5, OR52N1, OR52N2, OR52E6, OR52E8, OR52E4, OR56A3* |

**Table S3** Summary information of genomic regions with top 1% of Indel density over the genome.

| Chrom-osome | Start (Mb) | End (Mb) | # SNVs per Mb | Protein-coding Genes |
| --- | --- | --- | --- | --- |
| 6 | 29 | 33 | 910 | *OR5V1,OR11A1,UBD,GABBR1,MOG,ZFP57,HLA-F,HLA-G,HLA-A,ZNRD1,RNF39,TRIM31,TRIM40,TRIM10,TRIM15,TRIM26,TRIM39,TRIM39-RPP21,GNL1,PRR3,ABCF1,PPP1R10,MRPS18B,ATAT1,C6orf136,DHX16,MDC1,TUBB,FLOT1,IER3,DDR1,VARS2,DPCR1,MUC22,PSORS1C1,CDSN,CCHCR1,TCF19,POU5F1,HCG27,HLA-C,HLA-B,MICA,MICB,MCCD1,DDX39B,ATP6V1G2-DDX39B,ATP6V1G2,NFKBIL1,LTA,LST1,NCR3,PRRC2A,BAG6,C6orf47,GPANK1,CSNK2B,CSNK2B-LY6G5B-1181,LY6G5B,LY6G5C,ABHD16A,XXbac-BPG32J3.20,LY6G6F,MEGT1,LY6G6D,C6orf25,CLIC1,MSH5,MSH5-SAPCD1,SAPCD1,VWA7,VARS,LSM2,HSPA1L,HSPA1B,C6orf48,SLC44A4,EHMT2,C2,ZBTB12,CFB,NELFE,SKIV2L,DXO,STK19,CYP21A2,TNXB,ATF6B,FKBPL,PPT2,PPT2-EGFL8,EGFL8,AGPAT1,RNF5,AGER,PBX2,NOTCH4,C6orf10,BTNL2,HLA-DRA,HLA-DRB5,HLA-DRB1,HLA-DQA1,HLA-DQB1,HLA-DQA2,HLA-DQB2,HLA-DOB,TAP2,PSMB9,TAP1,HLA-DMB,XXbac-BPG181M17.5,HLA-DMA,BRD2,HLA-DOA* |
| 19 | 7 | 8 | 717 | *AC025278.1,MBD3L5,MBD3L2,ZNF557,INSR,CTB-133G6.1,CTD-2207O23.3,ARHGEF18,PEX11G,C19orf45,CTD-2207O23.12,ZNF358,MCOLN1,PNPLA6,CAMSAP3,XAB2,CTD-3214H19.4,PCP2,STXBP2,C19orf59,FCER2,CLEC4G,CD209,EVI5L,CTD-3193O13.9,LRRC8E,AC010336.1,MAP2K7,TIMM44* |
| 8 | 3 | 4 | 705 | *CSMD1* |
| 19 | 52 | 55 | 693 | *SIGLEC12,SIGLEC6,ZNF175,AC018755.1,SIGLEC5,SIGLEC14,HAS1,FPR1,FPR2,FPR3,ZNF577,ZNF649,ZNF613,ZNF350,ZNF615,ZNF614,ZNF432,ZNF841,ZNF616,ZNF836,PPP2R1A,ZNF766,ZNF480,ZNF610,ZNF880,ZNF528,ZNF534,ZNF578,ZNF808,ZNF701,ZNF83,ZNF611,ZNF600,ZNF28,ZNF468,ZNF320,ZNF816,ZNF321P,ERVV-1,ERVV-2,ZNF160,ZNF415,ZNF347,ZNF665,ZNF677,ZNF845,ZNF525,ZNF765,ZNF813,ZNF331,CTB-167G5.5,DPRX,NLRP12,MYADM,PRKCG,CACNG7,CACNG8,CACNG6,VSTM1,TARM1,OSCAR,NDUFA3,TFPT,PRPF31,CNOT3,TMC4,MBOAT7,TSEN34,RPS9,LILRB3,LILRA6,LILRB5,LILRB2,LILRA3,LILRA5,LILRA4,LAIR1,TTYH1,LENG8,LENG9,CDC42EP5* |
| 7 | 66 | 68 | 692 | *KCTD7,RABGEF1,TMEM248,SBDS,TYW1* |
| 12 | 32 | 33 | 684 | *KIAA1551,BICD1,FGD4,DNM1L,YARS2,PKP2* |
| 1 | 236 | 237 | 680 | *LYST,NID1,GPR137B,ERO1LB,EDARADD,LGALS8,HEATR1,ACTN2,MTR* |
| 4 | 39 | 40 | 669 | *TMEM156,KLHL5,WDR19,RFC1,KLB,RPL9,LIAS,UGDH,SMIM14,UBE2K,PDS5A* |
| 22 | 49 | 50 | 667 | *FAM19A5,C22orf34* |
| 7 | 5 | 6 | 664 | *RBAK-RBAKDN,RBAK,WIPI2,SLC29A4,TNRC18,FBXL18,ACTB,FSCN1,RNF216,OCM,CCZ1,RSPH10B* |
| 4 | 189 | 190 | 655 | *TRIML2,TRIML1* |
| 21 | 27 | 28 | 645 | *JAM2,ATP5J,GABPA,APP,CYYR1* |
| *(Continued)* | | | | |
| 10 | 27 | 28 | 643 | *PDSS1,ABI1,ANKRD26,YME1L1,MASTL,ACBD5,PTCHD3,RAB18,MKX* |
| 19 | 56 | 57 | 643 | *SSC5D,SBK2,SBK3,ZNF579,FIZ1,ZNF524,ZNF580,ZNF581,CCDC106,U2AF2,EPN1,NLRP9,RFPL4A,RFPL4AL1,NLRP11,NLRP4,NLRP13,NLRP8,NLRP5,ZNF787,ZNF444,GALP,ZSCAN5B,ZSCAN5C,ZSCAN5A,ZSCAN5D,AC006116.20,ZNF582,ZNF583,ZNF667* |
| 16 | 12 | 13 | 635 | *GSPT1,RP11-166B2.1,TNFRSF17,SNX29,RP11-276H1.3,CPPED1,SHISA9* |
| 12 | 95 | 96 | 633 | *TMCC3,NDUFA12,NR2C1,FGD6,VEZT,RP11-167N24.6,METAP2,USP44* |
| 11 | 24 | 25 | 630 | *LUZP2* |
| 12 | 121 | 122 | 624 | *RNF10,POP5,CABP1,MLEC,UNC119B,ACADS,SPPL3,HNF1A,C12orf43,OASL,P2RX7,P2RX4,CAMKK2,ANAPC5,RNF34,KDM2B* |
| 2 | 1 | 2 | 622 | *SNTG2,TPO,PXDN,MYT1L* |
| 17 | 12 | 13 | 617 | *MAP2K4,MYOCD,AC005358.1,ARHGAP44,ELAC2* |

**Table S4** Functional enrichment of genes underlying the mutation hotspots, loss-of-function variants, *de novo* variants, copy number variants and novel insertions.

Significantly enriched categories are highlighted in red.

(see Additional file 2.xlsx)

**Table S5** Functional annotation of SNVs in each native population and global populations

| Population | # Samples | # SNVs (Mb) | % Novelty | Het:Hom | Variant Impact | | | |  | Variant Type | | | |  |
| --- | --- | --- | --- | --- | --- | --- | --- | --- | --- | --- | --- | --- | --- | --- |
|  |  |  |  |  | High | Moderate | Low | Modifier |  | LOF | Syn | Non-syn | Syn:Non-syn |  |
| *Southeast Asia* | | | | | | | | | | | | |  |  |
| Bateq | 3 | 3.31 | 1.17 | 1.15 | 890 | 13,759 | 35,371 | 3,264,393 |  | 275 | 11,436 | 11,003 | 1.04 |  |
| Mendriq | 3 | 3.36 | 0.84 | 1.26 | 912 | 13,912 | 36,067 | 3,310,365 |  | 298 | 11,480 | 11,055 | 1.04 |  |
| Semai | 3 | 3.33 | 0.68 | 1.19 | 920 | 13,948 | 35,540 | 3,276,085 |  | 287 | 11,510 | 11,093 | 1.04 |  |
| Murut | 3 | 3.31 | 0.32 | 1.17 | 908 | 13,594 | 35,435 | 3,260,865 |  | 276 | 11,228 | 10,907 | 1.03 |  |
| Dusun | 3 | 3.32 | 0.33 | 1.18 | 913 | 13,761 | 35,855 | 3,271,365 |  | 296 | 11,417 | 10,921 | 1.04 |  |
| SSM | 96 | 3.39 | - | 1.38 | 953 | 14,321 | 35,967 | 3,339,107 |  | 293 | 11,842 | 11,523 | 1.03 |  |
| KHV | 99 | 3.53 | - | 1.33 | 964 | 14,594 | 37,126 | 3,473,730 |  | 308 | 10,408 | 9,605 | 1.08 |  |
| *East Asia* | | | | | | | | | | | | |  |  |
| CDX | 99 | 3.51 | - | 1.30 | 961 | 14,595 | 37,050 | 3,458,641 |  | 307 | 10,380 | 9,583 | 1.08 |  |
| CHS | 105 | 3.51 | - | 1.31 | 960 | 14,566 | 36,943 | 3,457,734 |  | 310 | 10,401 | 9,596 | 1.08 |  |
| CHB | 103 | 3.52 | - | 1.32 | 964 | 14,609 | 37,028 | 3,466,103 |  | 310 | 10,428 | 9,636 | 1.08 |  |
| JPT | 104 | 3.52 | - | 1.31 | 958 | 14,644 | 36,829 | 3,462,745 |  | 310 | 10,399 | 9,611 | 1.08 |  |
| *South Asia* | | | | | | | | | | | | |  |  |
| SSI | 36 | 3.20 | - | 1.51 | 866 | 13,059 | 34,670 | 3,149,371 |  | 270 | 11,104 | 10,298 | 1.08 |  |
| BEB | 86 | 3.61 | - | 1.58 | 975 | 14,959 | 38,286 | 3,550,976 |  | 307 | 10,671 | 9,812 | 1.09 |  |
| GIH | 103 | 3.58 | - | 1.56 | 972 | 14,820 | 37,942 | 3,530,425 |  | 303 | 10,624 | 9,753 | 1.09 |  |
| PJL | 96 | 3.58 | - | 1.54 | 962 | 14,817 | 37,933 | 3,523,034 |  | 306 | 10,603 | 9,749 | 1.09 |  |
| ITU | 102 | 3.59 | - | 1.53 | 967 | 14,850 | 37,964 | 3,533,566 |  | 305 | 10,598 | 9,729 | 1.09 |  |
| STU | 102 | 3.58 | - | 1.52 | 967 | 14,839 | 38,001 | 3,530,389 |  | 305 | 10,572 | 9,729 | 1.09 |  |
| *(Continued)* | | | | | | | | | | | | | |  |
| *Europe* | | | | | | | | | | | | | | |
| FIN | 99 | 3.51 | - | 1.53 | 946 | 14,526 | 37,162 | 3,452,924 |  | 295 | 10,428 | 9,597 | 1.09 |  |
| GBR | 91 | 3.51 | - | 1.54 | 948 | 14,538 | 37,338 | 3,455,564 |  | 296 | 10,384 | 9,571 | 1.09 |  |
| CEU | 99 | 3.51 | - | 1.55 | 947 | 14,503 | 37,300 | 3,461,288 |  | 293 | 10,391 | 9,557 | 1.09 |  |
| IBS | 107 | 3.53 | - | 1.56 | 954 | 14,625 | 37,469 | 3,477,724 |  | 296 | 10,408 | 9,585 | 1.09 |  |
| TSI | 107 | 3.53 | - | 1.56 | 956 | 14,655 | 37,579 | 3,478,046 |  | 299 | 10,451 | 9,632 | 1.09 |  |
| *America* | | | | | | | | | | | | |  |  |
| MXL | 64 | 3.57 | - | 1.53 | 953 | 14,705 | 37,763 | 3,517,521 |  | 303 | 10,463 | 9,586 | 1.09 |  |
| CLM | 94 | 3.63 | - | 1.65 | 979 | 14,999 | 38,381 | 3,573,844 |  | 308 | 10,553 | 9,672 | 1.09 |  |
| PUR | 104 | 3.69 | - | 1.73 | 1,002 | 15,281 | 39,113 | 3,632,839 |  | 315 | 10,601 | 9,707 | 1.09 |  |
| PEL | 85 | 3.49 | - | 1.32 | 929 | 14,361 | 36,710 | 3,442,879 |  | 301 | 10,252 | 9,381 | 1.09 |  |
| *Africa* | | | | | | | | | | | | |  |  |
| YRI | 108 | 4.29 | - | 1.93 | 1,175 | 17,783 | 45,811 | 4,225,843 |  | 362 | 10,585 | 9,440 | 1.12 |  |
| GWD | 113 | 4.28 | - | 1.92 | 1,166 | 17,640 | 45,741 | 4,214,771 |  | 360 | 10,571 | 9,432 | 1.12 |  |
| MSL | 85 | 4.34 | - | 1.96 | 1,186 | 17,911 | 46,449 | 4,273,288 |  | 363 | 10,603 | 9,427 | 1.12 |  |
| ESN | 99 | 4.29 | - | 1.93 | 1,166 | 17,777 | 45,873 | 4,229,002 |  | 362 | 10,582 | 9,443 | 1.12 |  |
| LWK | 99 | 4.29 | - | 1.97 | 1,167 | 17,730 | 45,768 | 4,226,605 |  | 361 | 10,662 | 9,511 | 1.12 |  |
| ACB | 96 | 4.26 | - | 2.02 | 1,161 | 17,588 | 45,505 | 4,191,093 |  | 358 | 10,685 | 9,573 | 1.12 |  |
| ASW | 61 | 4.19 | - | 2.06 | 1,145 | 17,274 | 44,671 | 4,126,898 |  | 352 | 10,744 | 9,637 | 1.12 |  |

The number of SNVs in each category and the ratio of heterozygotes over homozygotes (Het:Hom) were calculated as the average across individuals in each population. Only autosomal SNVs were included.

**Table S6** Functional annotation of indels in each native population and global populations

| Population | # Samples | # Indel | % Novelty | Variant Impact | | | | |
| --- | --- | --- | --- | --- | --- | --- | --- | --- |
|  |  |  |  | High | Moderate | Low | Modifier | LOF |
| *Southeast Asia* | | | | | | | | |
| Bateq | 3 | 616,543 | 1.41 | 481 | 943 | 5,434 | 609,685 | 314 |
| Mendriq | 3 | 639,091 | 1.26 | 456 | 961 | 5,704 | 631,969 | 293 |
| Semai | 3 | 618,573 | 1.20 | 477 | 960 | 5,477 | 611,659 | 308 |
| Murut | 3 | 616,296 | 1.01 | 482 | 953 | 5,494 | 609,367 | 321 |
| Dusun | 3 | 616,692 | 1.02 | 462 | 903 | 5,475 | 609,851 | 302 |
| SSM | 96 | 507,968 | - | 358 | 600 | 3,860 | 440,502 | 241 |
| KHV | 99 | 508,216 | - | 425 | 770 | 4,536 | 502,484 | 270 |
| *East Asia* | | | | | | | | |
| CDX | 93 | 505,310 | - | 422 | 763 | 4,519 | 499,604 | 268 |
| CHS | 105 | 505,816 | - | 422 | 769 | 4,514 | 500,109 | 268 |
| CHB | 103 | 505,697 | - | 424 | 769 | 4,508 | 499,995 | 269 |
| JPT | 104 | 507,988 | - | 425 | 772 | 4,515 | 502,275 | 269 |
| *South Asia* | | | | | | | | |
| SSI | 36 | 375,734 | - | 367 | 554 | 3,214 | 371,597 | 255 |
| BEB | 86 | 516,307 | - | 435 | 788 | 4,634 | 510,449 | 279 |
| GIH | 103 | 512,895 | - | 434 | 777 | 4,591 | 507,093 | 278 |
| PJL | 96 | 514,371 | - | 436 | 784 | 4,622 | 508,528 | 280 |
| ITU | 102 | 514,769 | - | 435 | 783 | 4,614 | 508,935 | 279 |
| STU | 102 | 514,576 | - | 436 | 782 | 4,625 | 508,731 | 279 |
| *Europe* | | | | | | | | |
| FIN | 99 | 504,301 | - | 428 | 764 | 4,531 | 498,576 | 273 |
| GBR | 91 | 505,037 | - | 425 | 768 | 4,556 | 499,287 | 273 |
| CEU | 99 | 505,874 | - | 424 | 769 | 4,554 | 500,125 | 270 |
| IBS | 107 | 506,927 | - | 424 | 780 | 4,564 | 501,158 | 272 |
| TSI | 107 | 507,473 | - | 427 | 771 | 4,575 | 501,698 | 274 |
| *America* | | | | | | | | |
| MXL | 64 | 513,341 | - | 427 | 776 | 4,611 | 507,526 | 270 |
| CLM | 94 | 518,520 | - | 435 | 788 | 4,641 | 512,654 | 277 |
| PUR | 104 | 523,866 | - | 439 | 794 | 4,691 | 517,940 | 281 |
| *(Continued)* | | | | | | | | |
| PEL | 85 | 503,911 | - | 418 | 762 | 4,492 | 498,237 | 265 |
| *Africa* | | | | | | | | |
| YRI | 108 | 575,926 | - | 495 | 872 | 5,142 | 569,415 | 326 |
| GWD | 113 | 577,027 | - | 486 | 866 | 5,190 | 570,484 | 318 |
| MSL | 85 | 579,714 | - | 497 | 874 | 5,208 | 573,133 | 329 |
| ESN | 99 | 577,009 | - | 494 | 874 | 5,162 | 570,477 | 326 |
| LWK | 99 | 574,708 | - | 486 | 863 | 5,137 | 568,220 | 320 |
| ACB | 96 | 573,496 | - | 489 | 867 | 5,138 | 567,000 | 321 |
| ASW | 61 | 567,831 | - | 482 | 856 | 5,078 | 561,414 | 314 |

The number of indels in each category were calculated as the average across individuals in each population. Only autosomal indels were included.

**Table S7** Functional annotation of SNVs and indels in each native Malaysian genome

| Sample | # SNVs | | | | | |  | # indels | | | | | |
| --- | --- | --- | --- | --- | --- | --- | --- | --- | --- | --- | --- | --- | --- |
|  | Total | High | Moderate | Low | Modifier | LOF |  | Total | High | Moderate | Low | Modifier | LOF |
| *Bateq* | | | | | | |  |  |  |  |  |  |  |
| BTQ016 | 3,414,563 | 913 | 14,066 | 36,035 | 3,363,549 | 291 |  | 640,246 | 501 | 975 | 5,603 | 633,167 | 334 |
| BTQ055 | 3,422,095 | 903 | 13,846 | 35,998 | 3,371,348 | 260 |  | 640,858 | 505 | 923 | 5,576 | 633,854 | 328 |
| BTQ038 | 3,389,212 | 901 | 14,021 | 35,788 | 3,338,502 | 283 |  | 638,264 | 484 | 980 | 5,588 | 631,212 | 320 |
| *Mendriq* | | | | | | |  |  |  |  |  |  |  |
| MDQ045 | 3,452,177 | 921 | 14,043 | 36,535 | 3,400,678 | 283 |  | 663,040 | 472 | 979 | 5,814 | 655,775 | 308 |
| MDQ025 | 3,438,669 | 944 | 14,052 | 36,346 | 3,387,327 | 310 |  | 657,903 | 465 | 954 | 5,819 | 650,665 | 297 |
| MDQ010 | 3,498,938 | 928 | 14,405 | 37,136 | 3,446,469 | 313 |  | 672,629 | 477 | 1,005 | 5,947 | 665,200 | 314 |
| *Semai* | | | | | | |  |  |  |  |  |  |  |
| SMI018 | 3,419,734 | 935 | 14,336 | 36,412 | 3,368,051 | 286 |  | 640,281 | 501 | 973 | 5,628 | 633,179 | 327 |
| SMI034 | 3,442,291 | 950 | 14,232 | 36,258 | 3,390,851 | 299 |  | 645,695 | 489 | 992 | 5,652 | 638,562 | 316 |
| SMI041 | 3,389,552 | 931 | 13,944 | 35,545 | 3,339,132 | 292 |  | 637,827 | 483 | 975 | 5,568 | 630,801 | 315 |
| *Murut* | | | | | | |  |  |  |  |  |  |  |
| NB10 | 3,394,575 | 931 | 13,820 | 36,050 | 3,343,774 | 295 |  | 636,066 | 485 | 934 | 5,531 | 629,116 | 325 |
| NB11 | 3,443,476 | 939 | 14,130 | 36,404 | 3,392,003 | 313 |  | 644,162 | 509 | 1,001 | 5,664 | 636,988 | 341 |
| NB12 | 3,407,902 | 923 | 14,016 | 36,741 | 3,356,222 | 289 |  | 638,245 | 496 | 989 | 5,697 | 631,063 | 334 |
| *Dusun* | | | | | | |  |  |  |  |  |  |  |
| NB07 | 3,396,196 | 941 | 13,789 | 35,829 | 3,345,637 | 277 |  | 638,513 | 475 | 945 | 5,596 | 631,497 | 310 |
| NB08 | 3,412,977 | 900 | 13,708 | 35,966 | 3,362,403 | 271 |  | 640,842 | 462 | 902 | 5,617 | 633,861 | 310 |
| NB09 | 3,400,300 | 928 | 13,960 | 36,150 | 3,349,262 | 287 |  | 640,086 | 488 | 930 | 5,629 | 633,039 | 321 |

Variants in the chromosomes 1-22, X and Y, and mitochondrial DNA are all included.

**Table S8** Distribution of FS indels and the candidate genes affected

(see Additional file 3.xlsx)

**Table S9** Genomic regions identified as novel SNV hotspots

| Chromosome | Start (Mb) | End (Mb) | # SNV per Mb | # Coding genes |
| --- | --- | --- | --- | --- |
| 2 | 51 | 52 | 135 | 1 |
| 2 | 207 | 208 | 148 | 8 |
| 2 | 217 | 218 | 133 | 6 |
| 3 | 2 | 3 | 131 | 1 |
| 4 | 43 | 44 | 127 | 1 |
| 5 | 19 | 21 | 448 | 1 |
| 5 | 27 | 28 | 240 | 1 |
| 6 | 67 | 68 | 122 | 0 |
| 6 | 164 | 165 | 132 | 0 |
| 7 | 3 | 4 | 174 | 3 |
| 7 | 23 | 24 | 125 | 10 |
| 8 | 3 | 6 | 449 | 1 |
| 8 | 13 | 15 | 251 | 2 |
| 8 | 18 | 19 | 125 | 3 |
| 9 | 22 | 23 | 128 | 3 |
| 11 | 55 | 56 | 126 | 5 |
| 11 | 81 | 82 | 130 | 0 |
| 12 | 38 | 39 | 137 | 0 |
| 15 | 90 | 91 | 122 | 20 |
| 16 | 78 | 79 | 137 | 3 |
| 16 | 83 | 85 | 266 | 19 |

**Table S10** Genomic regions identified as novel indel hotspots

| Chromosome | Start (Mb) | End (Mb) | # Indels per Mb | # Coding genes |
| --- | --- | --- | --- | --- |
| 1 | 1 | 2 | 27 | 8 |
| 1 | 12 | 14 | 51 | 7 |
| 1 | 145 | 148 | 156 | 22 |
| 1 | 149 | 151 | 54 | 8 |
| 3 | 60 | 61 | 26 | 1 |
| 5 | 19 | 21 | 53 | 1 |
| 5 | 27 | 28 | 28 | 1 |
| 7 | 72 | 73 | 43 | 6 |
| 7 | 142 | 143 | 37 | 2 |
| 8 | 2 | 3 | 53 | 2 |
| 8 | 117 | 118 | 23 | 3 |
| 12 | 11 | 12 | 27 | 5 |
| 14 | 22 | 23 | 94 | 0 |
| 17 | 34 | 37 | 132 | 22 |
| 19 | 8 | 9 | 30 | 4 |
| 19 | 42 | 43 | 34 | 8 |
| 19 | 45 | 46 | 32 | 5 |
| 19 | 54 | 55 | 43 | 16 |
| 21 | 46 | 47 | 27 | 9 |
| 22 | 22 | 23 | 60 | 5 |
| 22 | 49 | 50 | 25 | 2 |

**Table S11** List of the *de novo* SNVs identified in each offspring

| Chrom-osome | | SNP ID | Position | Parental Allele | | Mutant Allele | | MQ | | DP_offspring_ | DP_father_ | | DP_mother_ | | Gene |
| --- | --- | --- | --- | --- | --- | --- | --- | --- | --- | --- | --- | --- | --- | --- | --- |
| *Bateq (BTQ038)* | | | | | | | | | | | | | | |  |
| 1 | | NA | 118556131 | G | | T | | 59.9 | | 56 | 30 | | 41 | | *SPAG17* |
| 1 | | NA | 154847735 | G | | T | | 59 | | 31 | 33 | | 40 | | *-* |
| 2 | | NA | 59670863 | T | | C | | 59.7 | | 41 | 40 | | 39 | | *RP11-444A22.1, AC007131.2* |
| 2 | | rs201080172 | 109858570 | T | | A | | 57.8 | | 33 | 21 | | 34 | | *SH3RF3* |
| 2 | | NA | 123244067 | T | | C | | 59.9 | | 51 | 39 | | 53 | | *-* |
| 2 | | rs369541564 | 145158740 | C | | T | | 59.8 | | 52 | 40 | | 41 | | *ZEB2* |
| 3 | | rs145414915 | 5192151 | A | | G | | 52.3* | | 38 | 33 | | 29 | | *ARL8B* |
| 3 | | rs1024126960 | 10048761 | G | | A | | 59.6 | | 32 | 38 | | 40 | | *EMC3, AC022007.5* |
| 3 | | NA | 33782162 | G | | T | | 58.5 | | 38 | 45 | | 33 | | *-* |
| 3 | | NA | 89762872 | A | | G | | 59.7 | | 40 | 38 | | 38 | | *-* |
| 3 | | rs114671477 | 163243407 | C | | A | | 60.1 | | 36 | 44 | | 24 | | *-* |
| 3 | | NA | 165635446 | A | | C | | 59.1 | | 32 | 30 | | 36 | | *-* |
| 4 | | rs905062125 | 68422335 | T | | C | | 59.4 | | 35 | 41 | | 51 | | *-* |
| 4 | | rs1307869426 | 72035425 | G | | T | | 59.7 | | 26 | 29 | | 43 | | *-* |
| 4 | | NA | 119224462 | G | | C | | 59.8 | | 45 | 49 | | 52 | | *PRSS12* |
| 4 | | NA | 146814798 | C | | A | | 59.9 | | 41 | 43 | | 44 | | *ZNF827* |
| 4 | | rs201989739 | 154887467 | A | | C | | 58.9 | | 37 | 20 | | 38 | | *-* |
| 4 | | rs958372760 | 178420913 | G | | A | | 59.6 | | 26 | 45 | | 21 | | *RP11-130F10.1* |
| 4 | | rs543309047 | 189071107 | C | | T | | 59.3 | | 26 | 41 | | 24 | | *-* |
| 5 | | NA | 13322388 | T | | C | | 59.7 | | 43 | 27 | | 59 | | *-* |
| 5 | | rs1334827635 | 44155877 | C | | T | | 59.7 | | 45 | 35 | | 49 | | *-* |
| 5 | | rs917930991 | 84951050 | G | | A | | 59.6 | | 43 | 39 | | 32 | | *-* |
| 5 | | rs969462023 | 153239792 | T | | C | | 59.5 | | 27 | 37 | | 38 | | *-* |
| 6 | | NA | 87367634 | G | | T | | 54.3 | | 46 | 34 | | 32 | | *-* |
| 6 | | NA | 122107031 | G | | C | | 59.8 | | 38 | 44 | | 29 | | *-* |
| 6 | | NA | 133597757 | T | | C | | 58.1 | | 39 | 45 | | 50 | | *EYA4* |
| 7 | | NA | 4197300 | G | | A | | 54.2 | | 34 | 41 | | 38 | | *SDK1* |
| 7 | | rs528257366 | 148369839 | C | | G | | 59.4 | | 41 | 35 | | 32 | | *-* |
| 8 | | NA | 117990891 | A | | C | | 57.3 | | 48 | 46 | | 44 | | *SLC30A8* |
| 10 | | rs185589411 | 3152397 | A | | T | | 53.6 | | 36 | 30 | | 40 | | *PFKP* |
| 10 | | NA | 90864643 | C | | A | | 59.7 | | 56 | 37 | | 43 | | *-* |
| 10 | | rs1432820043 | 90923365 | T | | C | | 59.7 | | 36 | 37 | | 39 | | *-* |
| 10 | | NA | 93455525 | G | | T | | 59.5 | | 39 | 45 | | 33 | | *-* |
| 11 | | NA | 94861070 | C | | G | | 59.0 | | 42 | 36 | | 38 | | *ENDOD1* |
| 11 | | rs73579528 | 115646755 | G | | A | | 55.8 | | 29 | 35 | | 45 | | *AP000797.3* |
| 12 | | NA | 68361770 | A | | C | | 58.6 | | 36 | 36 | | 36 | | *GS1-410F4.4* |
| 12 | | rs966039855 | 98650314 | G | | A | | 59.8 | | 41 | 45 | | 42 | | *RP11-690J15.1* |
| 12 | | NA | 113647651 | C | | T | | 58.9 | | 41 | 41 | | 37 | | *IQCD* |
| 12 | | NA | 131422553 | T | | C | | 58.3 | | 47 | 41 | | 48 | | *-* |
| 13 | | rs1258319963 | 97111403 | G | | A | | 56.5 | | 26 | 28 | | 23 | | *HS6ST3* |
| 13 | | rs142322428 | 110338396 | C | | T | | 59.7 | | 55 | 43 | | 58 | | *-* |
| 14 | | NA | 59096409 | G | | T | | 58.7 | | 38 | 41 | | 40 | | *-* |
| 14 | | NA | 103483863 | C | | T | | 59.7 | | 42 | 44 | | 35 | | *CDC42BPB* |
| 15 | | NA | 39306165 | G | | A | | 59.7 | | 43 | 36 | | 38 | | *RP11-624L4.1* |
| 18 | | NA | 20175304 | A | | G | | 59.8 | | 45 | 48 | | 45 | | *-* |
| 18 | | NA | 28145149 | C | | T | | 59.2 | | 48 | 33 | | 39 | | *-* |
| 18 | | rs78888408 | 33103672 | C | | T | | 56.8* | | 24 | 28 | | 18 | | *-* |
| 20 | | NA | 52970880 | T | | A | | 59.8 | | 41 | 36 | | 30 | | *-* |
| 22 | | NA | 31462836 | G | | C | | 58.9 | | 46 | 33 | | 35 | | *SMTN* |
| *Mendriq (MDQ010)* | | | | | | | | | | | | | | |  |
| *(Continued)* | | | | | | | | | | | | | | |  |
| 1 | | NA | 195993792 | T | | C | | 59.7 | | 40 | 40 | | 26 | | *-* |
| 2 | | NA | 42972487 | G | | A | | 59.9 | | 29 | 21 | | 28 | | *MTA3* |
| 2 | | rs560811488 | 77795922 | C | | T | | 59.5 | | 37 | 40 | | 34 | | *LRRTM4* |
| 2 | | rs764855486 | 141474240 | A | | G | | 59.6 | | 31 | 52 | | 23 | | *LRP1B* |
| 2 | | NA | 176312742 | C | | G | | 59.9 | | 33 | 40 | | 37 | | *AC096649.3* |
| 2 | | rs915302384 | 177848080 | G | | A | | 59.6 | | 31 | 42 | | 26 | | *-* |
| 3 | | NA | 1273039 | T | | C | | 59.8 | | 36 | 38 | | 38 | | *CNTN6* |
| 3 | | NA | 13003155 | C | | A | | 59.7 | | 35 | 34 | | 34 | | *IQSEC1* |
| 3 | | NA | 22491979 | T | | C | | 59.8 | | 40 | 50 | | 31 | | *-* |
| 3 | | NA | 114923071 | G | | A | | 59.9 | | 45 | 48 | | 23 | | *-* |
| 4 | | rs1240503254 | 11469854 | C | | T | | 55.2 | | 28 | 32 | | 17 | | *-* |
| 5 | | rs1056026119 | 62972832 | G | | C | | 49.3 | | 46 | 30 | | 39 | | *-* |
| 5 | | rs1356951164 | 131435812 | C | | T | | 59.8 | | 40 | 36 | | 26 | | *-* |
| 5 | | rs1006281790 | 134185773 | A | | G | | 58.8 | | 46 | 41 | | 18 | | *DDX46, C5orf24* |
| 6 | | rs936709149 | 5816278 | G | | A | | 59.5 | | 34 | 30 | | 37 | | *-* |
| 7 | | NA | 4334135 | T | | C | | 59.6 | | 58 | 31 | | 52 | | *-* |
| 8 | | NA | 2310432 | G | | A | | 59.7* | | 29 | 32 | | 27 | | *-* |
| 8 | | NA | 41289952 | G | | C | | 59.7 | | 38 | 32 | | 34 | | *-* |
| 8 | | rs541246258 | 54468943 | T | | C | | 59.8 | | 36 | 43 | | 34 | | *-* |
| 8 | | NA | 72315724 | A | | G | | 59.7 | | 54 | 37 | | 47 | | *RP11-1102P16.1* |
| 8 | | NA | 128584921 | G | | T | | 59.5 | | 50 | 47 | | 30 | | *-* |
| 8 | | NA | 139535122 | T | | C | | 59.8 | | 37 | 26 | | 33 | | *-* |
| 10 | | NA | 28009134 | G | | A | | 58.8 | | 20 | 37 | | 25 | | *MKX* |
| 10 | | NA | 84692399 | T | | C | | 59.9 | | 47 | 42 | | 28 | | *NRG3* |
| 10 | | rs568077488 | 98750878 | T | | C | | 59.5 | | 38 | 35 | | 25 | | *-* |
| 12 | | NA | 7704432 | G | | T | | 46.7 | | 50 | 51 | | 35 | | *-* |
| 12 | | NA | 28342410 | C | | T | | 59.9 | | 39 | 47 | | 32 | | *CCDC91,*  *RP11-967K21.1* |
| 12 | | NA | 46314452 | T | | C | | 59.8 | | 33 | 48 | | 28 | | *SCAF11* |
| 12 | | NA | 130461014 | A | | G | | 59.5 | | 46 | 40 | | 37 | | *-* |
| 13 | | rs910182559 | 94346944 | G | | T | | 59.6 | | 49 | 35 | | 29 | | *GPC6* |
| 13 | | rs937878340 | 112908835 | G | | A | | 59.4 | | 27 | 24 | | 17 | | *-* |
| 14 | | rs72668090 | 34526480 | C | | T | | 38.2 | | 35 | 26 | | 19 | | *EGLN3,*  *EGLN3-AS1* |
| 14 | | rs578005306 | 50851505 | C | | T | | 58.0 | | 22 | 31 | | 19 | | *CDKL1* |
| 14 | | rs1566731274 | 88817588 | A | | G | | 59.8 | | 42 | 37 | | 43 | | *-* |
| 17 | | rs62071218 | 35789907 | T | | C | | 60.0 | | 36 | 19 | | 33 | | *TADA2A* |
| 18 | | rs1225048168 | 65841495 | A | | G | | 59.7 | | 45 | 39 | | 35 | | *-* |
| 19 | | NA | 45094041 | C | | A | | 59.6 | | 22 | 30 | | 36 | | *CEACAM22P* |
| *Semai (SMI041)* | | | | | | | | | | | | | | |  |
| 1 | | NA | 211681985 | C | | A | | 59.5 | | 32 | 27 | | 29 | | *-* |
| 2 | | NA | 32635347 | C | | G | | 59.6 | | 46 | 30 | | 36 | | *BIRC6* |
| 2 | | NA | 38490979 | C | | A | | 59.6 | | 38 | 41 | | 30 | | *-* |
| 2 | | rs1388358750 | 51929984 | C | | T | | 59.5 | | 41 | 38 | | 33 | | *AC007682.1* |
| 2 | | NA | 58484437 | G | | T | | 55.2 | | 39 | 26 | | 32 | | *-* |
| 2 | | NA | 116822204 | G | | T | | 59.7 | | 29 | 31 | | 26 | | *-* |
| 3 | | NA | 18827562 | T | | C | | 59.8 | | 51 | 56 | | 40 | | *AC144521.1* |
| 3 | | NA | 31070753 | A | | G | | 59.5 | | 36 | 34 | | 35 | | *-* |
| 3 | | NA | 37067957 | A | | G | | 59.2 | | 39 | 32 | | 28 | | *MLH1* |
| 3 | | NA | 59053883 | A | | G | | 59.8 | | 40 | 39 | | 48 | | *CTD-2185K10.1* |
| 4 | | rs978098500 | 11159545 | C | | T | | 59.8 | | 32 | 43 | | 33 | | *-* |
| 4 | | rs779432762 | 82323406 | G | | A | | 59.8 | | 28 | 39 | | 28 | | *-* |
| 4 | | rs975687965 | 127601846 | C | | T | | 59.8 | | 30 | 44 | | 37 | | *-* |
| 4 | | NA | 158249663 | A | | G | | 59.8 | | 32 | 43 | | 37 | | *GRIA2* |
| *(Continued)* | | | | | | | | | | | | | | |  |
| 4 | | NA | 171900466 | G | | C | | 58.0 | | 31 | 33 | | 30 | | *-* |
| 6 | | NA | 50644723 | T | | C | | 59.5 | | 36 | 39 | | 42 | | *-* |
| 6 | | rs1157508928 | 53878546 | T | | C | | 59.8 | | 47 | 48 | | 34 | | *MLIP* |
| 6 | | rs114652456 | 82564881 | T | | A | | 60.1 | | 45 | 47 | | 19 | | *RP11-379B8.1* |
| 7 | | NA | 17789517 | T | | C | | 59.9 | | 26 | 30 | | 23 | | *-* |
| 8 | | rs1339593477 | 76662111 | A | | G | | 59.8 | | 47 | 46 | | 42 | | *-* |
| 8 | | NA | 88911428 | A | | C | | 59.3 | | 38 | 38 | | 41 | | *-* |
| 8 | | rs754234991 | 121908749 | C | | T | | 59.7 | | 42 | 41 | | 32 | | *-* |
| 9 | | NA | 33674418 | C | | G | | 58.4 | | 33 | 23 | | 31 | | *PTENP1* |
| 9 | | NA | 36343180 | T | | A | | 59.8 | | 41 | 39 | | 48 | | *RNF38* |
| 10 | | NA | 49453287 | A | | C | | 59.5 | | 40 | 29 | | 33 | | *FRMPD2* |
| 10 | | NA | 76813343 | C | | T | | 59.8 | | 33 | 37 | | 24 | | *DUPD1* |
| 10 | | NA | 83572660 | G | | T | | 59.7 | | 36 | 42 | | 38 | | *-* |
| 12 | | rs113508885 | 10368918 | C | | T | | 57.1 | | 48 | 37 | | 36 | | *GABARAPL1* |
| 12 | | rs180798270 | 71409180 | G | | A | | 59.8 | | 34 | 44 | | 57 | | *CTD-2021H9.1* |
| 12 | | rs757892991 | 131409400 | C | | T | | 57.0 | | 31 | 32 | | 20 | | *-* |
| 13 | | rs571261429 | 38160596 | T | | C | | 59.8 | | 34 | 48 | | 48 | | *POSTN* |
| 14 | | NA | 85149358 | A | | C | | 59.7 | | 40 | 37 | | 55 | | *-* |
| 14 | | NA | 87152678 | G | | C | | 59.8 | | 33 | 49 | | 36 | | *-* |
| 16 | | NA | 4544018 | C | | T | | 57.4 | | 34 | 42 | | 21 | | *NMRAL1, HMOX2* |
| 16 | | rs899929645 | 58667912 | C | | T | | 59.1 | | 30 | 32 | | 36 | | *-* |
| 17 | | NA | 71408804 | C | | T | | 59.1 | | 33 | 27 | | 21 | | *SDK2* |
| 18 | | rs934942909 | 13579685 | A | | G | | 59.9 | | 39 | 49 | | | 39 | *LDLRAD4* |
| 18 | | rs1262891515 | 19985338 | G | | A | | 59.8 | | 33 | 35 | | | 38 | *-* |
| 20 | | NA | 21176246 | G | | A | | 59.7 | | 29 | 23 | | | 41 | *PLK1S1,*  *RP4-777D9.2* |
| 21 | | NA | 25663581 | T | | C | | 56.0 | | 24 | 38 | | | 31 | *-* |
| *Dusun (NB09)* | | | | | | | | | | | | | | |  |
| 1 | | rs78452876 | 246463221 | T | | G | | 56.5 | | 26 | 26 | | 28 | | *SMYD3* |
| 2 | | NA | 125187783 | G | | A | | 59.7 | | 27 | 34 | | 30 | | *CNTNAP5* |
| 2 | | NA | 141657309 | C | | G | | 59.8 | | 37 | 32 | | 47 | | *LRP1B* |
| 2 | | NA | 176745389 | T | | C | | 59.9 | | 43 | 41 | | 31 | | *-* |
| 2 | | NA | 185753542 | C | | T | | 59.9 | | 57 | 46 | 46 | | | *ZNF804A* |
| 2 | | NA | 239512784 | G | | A | | 59.8 | | 43 | 47 | 51 | | | *-* |
| 3 | | NA | 265990 | G | | A | | 59.8 | | 40 | 37 | 48 | | | *CHL1* |
| 3 | | rs925217206 | 159981621 | C | | T | | 59.4 | | 32 | 29 | | 33 | | *RP11-432B6.3, IFT80* |
| 3 | | NA | 167769476 | A | | G | | 59.6 | | 35 | 36 | | 43 | | *GOLIM4* |
| 4 | | rs1317928181 | 23734006 | G | | T | | 59.8 | | 51 | 41 | | 45 | | *RP11-380P13.1, RP11-380P13.2* |
| 4 | | rs1206280641 | 123908274 | A | | G | | 59.8 | | 44 | 44 | | 40 | | *SPATA5* |
| 4 | | rs191975105 | 130417620 | A | | G | | 59.4 | | 36 | 38 | | 28 | | *-* |
| 4 | | rs183283728 | 164182941 | G | | A | | 59.7 | | 44 | 30 | | 31 | | *-* |
| 5 | | NA | 13644792 | A | | C | | 59.8 | | 35 | 46 | | 30 | | *-* |
| 5 | | NA | 32409515 | A | | C | | 58.6 | | 41 | 42 | | 24 | | *ZFR* |
| 5 | | NA | 103406312 | A | | C | | 59.1 | | 47 | 37 | | 34 | | *-* |
| 5 | | rs757140118 | 158027502 | C | | T | | 59.2 | | 47 | 21 | | 38 | | *-* |
| 5 | | NA | 168438242 | G | | T | | 59.1 | | 29 | 40 | | 28 | | *SLIT3* |
| 6 | | rs4714327 | 40273242 | C | | T | | 50.5* | | 46 | 27 | | 44 | | *-* |
| 6 | | rs182513884 | 85547296 | G | | A | | 59.0 | | 34 | 27 | | 45 | | *-* |
| 6 | | NA | 117902661 | G | | A | | 59.9 | | 42 | 31 | | 38 | | *GOPC* |
| 6 | | NA | 124583670 | A | | G | | 59.8 | | 52 | 44 | | 41 | | *NKAIN2* |
| 6 | | rs1013739218 | 129989898 | G | | A | | 59.5 | | 31 | 40 | | 36 | | *ARHGAP18* |
| 7 | | NA | 80746883 | C | | G | | 59.7 | | 52 | 34 | | 49 | | *-* |
| *(Continued)* | | | | | | | | | | | | | | |  |
| 7 | | rs373382231 | 84165851 | G | | | A | 59.8 | | 44 | 35 | | 22 | | *AC003984.1* |
| 7 | | rs569955129 | 106933722 | G | | | C | 59.5 | | 35 | 38 | | 29 | | *COG5* |
| 7 | | rs755953657 | 132202932 | G | | | A | 59.7 | | 28 | 28 | | 30 | | *PLXNA4* |
| 8 | | NA | 15371552 | C | | | T | 59.8 | | 48 | 35 | | 33 | | *TUSC3* |
| 8 | | NA | 15896324 | C | | | G | 59.8 | | 43 | 34 | | 30 | | *-* |
| 8 | | rs112505190 | 39141385 | C | | | T | 58.8 | | 33 | 24 | | 21 | | *ADAM32* |
| 8 | | NA | 74136646 | A | | | G | 59.6 | | 41 | 44 | | 33 | | *-* |
| 9 | | NA | 20167836 | A | | | G | 59.7 | | 33 | 41 | | 33 | | *-* |
| 9 | | NA | 31194429 | C | | | T | 59.7 | | 43 | 40 | | 40 | | *-* |
| 9 | | rs889455440 | 36992955 | G | | | A | 59.5 | | 40 | 48 | | 40 | | *PAX5* |
| 10 | | NA | 13039839 | C | | | T | 59.8 | | 37 | 27 | | 29 | | *CCDC3* |
| 10 | | rs959550517 | 93005496 | C | | | T | 59.5 | | 48 | 35 | | 44 | | *PCGF5* |
| 11 | | rs766493565 | 21251992 | A | | | G | 59.8 | | 41 | 37 | | 40 | | *NELL1* |
| 12 | | rs910823887 | 115747763 | G | | | A | 59.1 | | 27 | 22 | | 26 | | *-* |
| 13 | | NA | 74848217 | G | | | A | 59.9 | | 49 | 23 | | 38 | | *-* |
| 13 | | rs77999608 | 104112713 | A | | | T | 46.6 | | 28 | 27 | | 22 | | *-* |
| 14 | | NA | 36782893 | T | | | C | 59.9 | | 61 | 51 | | 43 | | *MBIP* |
| 14 | | NA | 87941890 | A | | | G | 59.9 | | 42 | 35 | | 39 | | *RP11-594C13.1* |
| 14 | | NA | 95539625 | T | | | G | 59.7 | | 30 | 37 | | 32 | | *-* |
| 15 | | NA | 83792775 | C | | | G | 59.9 | | 46 | 48 | | 61 | | *TM6SF1, HDGFRP3* |
| 16 | | rs889060856 | 27310707 | C | | | T | 58.8 | | 38 | 35 | | 53 | | *-* |
| 16 | | rs4468607 | 60120728 | T | | | A | 52.0* | | 31 | 36 | | 22 | | *-* |
| 16 | | NA | 86926753 | T | | | C | 60.0 | | 39 | 30 | | 33 | | *-* |
| 17 | | NA | 15383545 | G | | | C | 59.8 | | 43 | 43 | | 40 | | *CDRT4, TVP23C* |
| 17 | | NA | 61905918 | C | | | G | 59.7 | | 44 | 52 | | 35 | | *FTSJ3, PSMC5* |
| 18 | | NA | 31159635 | A | | | G | 59.7 | | 37 | 41 | | 44 | | *ASXL3* |
| 19 | | rs62108907 | 52244620 | A | | | T | 56.0* | | 20 | 25 | | 21 | | *-* |
| 20 | | NA | 38181652 | G | | | A | 59.8 | | 37 | 31 | | 41 | | *-* |
| 22 | | NA | 28567014 | A | | | C | 59.2 | | 37 | 42 | | 31 | | *TTC28* |
| 22 | | NA | 39952578 | A | | | G | 59.8 | | 33 | 35 | | 20 | | *-* |
| *Murut (NB12)* | | | | | | | | | | | | | | |  |
| 1 | | rs61477336 | 87718975 | T | A | | | | 57.4 | 48 | 44 | | 29 | | *-* |
| 1 | | NA | 92979491 | G | A | | | | 59.6 | 33 | 33 | | 42 | | *EVI5* |
| 1 | | NA | 121246837 | A | G | | | | 59.7 | 53 | 38 | | 48 | | *RP11-344P13.4* |
| 1 | | rs543164721 | 218864514 | C | T | | | | 59.8 | 47 | 24 | | 38 | | *-* |
| 1 | | rs902287826 | 233148320 | G | A | | | | 59.7 | 40 | 36 | | 23 | | *PCNXL2* |
| 2 | | NA | 51313281 | G | C | | | | 59.7 | 35 | 46 | | 39 | | *AC007682.1* |
| 2 | | rs1283815678 | 52869613 | C | T | | | | 55.0 | 29 | 29 | | 31 | | *-* |
| 2 | | NA | 230574458 | G | C | | | | 59.7 | 43 | 30 | | 36 | | *DNER* |
| 2 | | rs981979420 | 236493650 | C | T | | | | 59.0 | 32 | 30 | | 27 | | *AGAP1* |
| 3 | | NA | 30003330 | G | A | | | | 59.6 | 28 | 26 | | 31 | | *RBMS3* |
| 3 | | rs1313952623 | 122948663 | C | T | | | | 57.3 | 32 | 26 | | 26 | | *SEC22A* |
| 4 | | NA | 26276650 | A | C | | | | 59.6 | 35 | 48 | | 47 | | *RBPJ* |
| 4 | | rs939384955 | 77882771 | C | T | | | | 59.9 | 45 | 28 | | 44 | | *SEPT11* |
| 4 | | rs906394958 | 140292288 | G | A | | | | 59.4 | 38 | 49 | | 43 | | *NAA15* |
| 4 | | rs1443895740 | 159871564 | T | C | | | | 59.6 | 43 | 35 | | 30 | | *C4orf45* |
| 5 | | NA | 66692682 | C | A | | | | 59.9 | 45 | 33 | | 34 | | *RP11-434D9.1* |
| 5 | | NA | 167332203 | C | G | | | | 59.8 | 45 | 48 | | 45 | | *TENM2* |
| 6 | | rs200661462 | 99557707 | T | A | | | | 60.0 | 47 | 42 | | 25 | | *-* |
| 6 | | rs1013847298 | 152452866 | C | T | | | | 59.9 | 30 | 30 | | 27 | | *SYNE1* |
| 6 | | NA | 154960945 | G | A | | | | 59.8 | 31 | 50 | | 44 | | *-* |
| 6 | | rs1017532210 | 168248596 | C | T | | | | 59.9 | 41 | 40 | | 48 | | *MLLT4* |
| 7 | | rs1217339184 | 91873256 | T | C | | | | 59.9 | 42 | 38 | | 45 | | *KRIT1* |
| *(Continued)* | | | | | | | | | | | | | | |  |
| 7 | | NA | 151133028 | G | | A | | 59.9 | | 34 | 28 | | 39 | | *RP4-555L14.4, CRYGN* |
| 8 | | rs1450863898 | 9688348 | C | | G | | 59.6 | | 26 | 31 | | 42 | | *-* |
| 8 | | NA | 27219522 | C | | T | | 59.4 | | 45 | 26 | | 34 | | *PTK2B* |
| 8 | | NA | 34692361 | A | | C | | 59.5 | | 19 | 42 | | 27 | | *RP11-258J10.1* |
| 8 | | NA | 111537560 | A | | G | | 59.8 | | 55 | 49 | | 61 | | *-* |
| 9 | | NA | 9671967 | A | | G | | 59.8 | | 40 | 60 | | 44 | | *PTPRD* |
| 10 | | NA | 11013597 | T | | C | | 59.5 | | 28 | 32 | | 33 | | *-* |
| 10 | | rs1459498856 | 32722982 | T | | C | | 58.8 | | 46 | 36 | | 47 | | *-* |
| 10 | | NA | 43265494 | T | | C | | 59.5 | | 31 | 41 | | 38 | | *-* |
| 10 | | NA | 64822720 | T | | A | | 59.7 | | 33 | 21 | | 31 | | *-* |
| 10 | | rs923742403 | 106174112 | G | | A | | 59.6 | | 32 | 24 | | 21 | | *CCDC147* |
| 10 | | NA | 110887422 | G | | A | | 59.6 | | 42 | 35 | | 43 | | *-* |
| 10 | | rs746224390 | 112147258 | C | | T | | 59.6 | | 35 | 33 | | 26 | | *-* |
| 12 | | NA | 2605335 | A | | C | | 59.9 | | 35 | 37 | | 40 | | *CACNA1C* |
| 12 | | NA | 2605336 | G | | C | | 59.9 | | 35 | 37 | | 47 | | *CACNA1C* |
| 12 | | NA | 42546271 | G | | A | | 58.8 | | 37 | 47 | | 39 | | *-* |
| 12 | | NA | 42546272 | C | | A | | 58.8 | | 36 | 47 | | 38 | | *-* |
| 12 | | rs984357267 | 70197238 | G | | A | | 57.3 | | 42 | 32 | | 27 | | *RAB3IP, AC025263.3* |
| 13 | | NA | 46915521 | C | | T | | 59.8 | | 37 | 37 | | 47 | | *-* |
| 13 | | rs887634254 | 51755541 | C | | T | | 59.8 | | 45 | 37 | | 35 | | *-* |
| 14 | | rs28766370 | 74839583 | G | | A | | 56.8 | | 24 | 23 | | 36 | | *-* |
| 14 | | rs375750588 | 96512560 | G | | A | | 59.7 | | 34 | 28 | | 33 | | *C14orf132* |
| 16 | | rs951007496 | 26999722 | G | | A | | 59.5 | | 33 | 30 | | 30 | | *-* |
| 16 | | NA | 50452056 | A | | G | | 58.0 | | 43 | 34 | | 44 | | *-* |
| 16 | | rs538473484 | 51801539 | C | | T | | 59.8 | | 25 | 31 | | 23 | | *RP11-7O14.1* |
| 16 | | NA | 66611673 | G | | A | | 59.7 | | 37 | 38 | | 40 | | *CKLF-CMTM1, CMTM1* |
| 16 | | rs1000768609 | 79081275 | G | | C | | 58.8 | | 42 | 54 | | 42 | | *WWOX* |
| 17 | | rs979629021 | 954932 | C | | T | | 58.7 | | 31 | 39 | | 19 | | *ABR* |
| 17 | | rs565891973 | 1511154 | C | | T | | 58.8 | | 27 | 21 | | 33 | | *SLC43A2* |
| 17 | | rs1429414319 | 1513958 | G | | T | | 58.8 | | 38 | 42 | | 31 | | *SLC43A2* |
| 17 | | rs947759879 | 71261623 | C | | T | | 59.6 | | 44 | 34 | | 29 | | *-* |
| 18 | | rs12955260 | 41564776 | A | | T | | 52.3* | | 34 | 27 | | 26 | | *-* |
| 18 | | rs77744174 | 48677705 | G | | C | | 53.6* | | 28 | 21 | | 17 | | *-* |
| 18 | | NA | 52419511 | C | | G | | 59.6 | | 26 | 43 | | 34 | | *RAB27B* |
| 18 | | NA | 55977527 | A | | G | | 59.9 | | 49 | 46 | | 56 | | *NEDD4L* |
| 19 | | rs755832841 | 2105821 | G | | A | | 59.7 | | 30 | 31 | | 29 | | *AP3D1* |
| 19 | | NA | 8307452 | A | | G | | 59.5 | | 36 | 43 | | 29 | | *CERS4* |
| 19 | | rs62137158 | 18952545 | C | | T | | 59.8 | | 42 | 26 | | 25 | | *UPF1* |
| 21 | | NA | 32861220 | G | | T | | 59.8 | | 33 | 41 | | 46 | | *TIAM1* |
| 21 | | rs57952230 | 47366644 | A | | G | | 59.0 | | 27 | 27 | | 26 | | *-* |

MQ, mapping quality, SNPs located in the simple repeats regions (downloaded from http://genome.ucsc.edu/) are indicated with asterisks; DP, read depth; NA, not available.

**Table S12** List of the *de novo* indels identified in each offspring

| Chrom-osome | | SNP ID | Position | Parental Allele | Mutant Allele | MQ | DP_offspring_ | DP_father_ | DP_mother_ | Gene | |
| --- | --- | --- | --- | --- | --- | --- | --- | --- | --- | --- | --- |
| *Bateq (BTQ038)* | | | | | | | | | |  |  |
| 1 | | NA | 110270237 | CTAAA | C | 59.4 | 48 | 40 | 43 | *GSTM5,*  *RP4-735C1.4* | |
| 8 | | NA | 125503997 | AC | A | 59.0 | 41 | 34 | 47 | *TATDN1* | |
| 11 | | rs1382317065 | 103162173 | TA | T | 55.8* | 27 | 26 | 26 | *DYNC2H1* | |
| 15 | | NA | 74318343 | A | AAG | 59.5 | 49 | 38 | 33 | *PML* | |
| 17 | | NA | 29945021 | AG | A | 59.6 | 38 | 29 | 52 | *RP1-41C23.1* | |
| *Mendriq (MDQ010)* | | | | | | | | | |  |  |
| 2 | | NA | 30884369 | TCTA | T | 59.6 | 38 | 40 | 23 | *-* | |
| 3 | | NA | 14483145 | TGTTCCTCGA | T | 59.3 | 26 | 26 | 25 | *SLC6A6* | |
| 8 | | rs143221913 | 21198684 | CCTCAAGTTTGCAATACCT | C | 57.6 | 49 | 28 | 26 | *-* | |
| 12 | | NA | 51898380 | CA | C | 59.7 | 40 | 48 | 38 | *SLC4A8* | |
| 15 | | NA | 31601543 | TTAAC | T | 59.4 | 32 | 38 | 18 | *-* | |
| 15 | | rs1299234610 | 98207314 | TAAAC | T | 59.7 | 60 | 33 | 37 | *-* | |
| 16 | | rs1407568933 | 26311602 | AC | A | 56.7 | 38 | 36 | 30 | *-* | |
| 18 | | NA | 984904 | T | TAG | 59.8 | 46 | 36 | 26 | *RP11-78F17.1* | |
| 18 | | rs142451262 | 43567463 | TGTAGCTCAGTCCACACTGAGATCAGGAATATGTAAGTG | T | 57.1 | 31 | 40 | 30 | *PSTPIP2* | |
| 20 | | NA | 39563011 | TA | T | 59.9 | 39 | 44 | 32 | *-* | |
| *Semai (SMI041)* | | | | | | | | | |  |  |
| 2 | | NA | 57,662,029 | TTTA | T | 59.6 | 37 | 44 | 28 | - | |
| 5 | | NA | 169471860 | TC | T | 59.5 | 30 | 22 | 26 | *DOCK2* | |
| 8 | | rs544602567 | 55714168 | GAGA | G | 59.7 | 33 | 39 | 38 | *RP11-56A10.1* | |
| 12 | | NA | 33147898 | CAG | C | 59.6 | 46 | 38 | 45 | *-* | |
| 17 | | rs528179714 | 66054077 | CAGAT | C | 58.5* | 46 | 33 | 30 | *-* | |
| 19 | | rs34725065 | 22896442 | C | CT | 58.4 | 13 | 18 | 27 | *CTC-457E21.9* | |
| *Dusun (NB09)* | | | | | | | | | |  |  |
| 4 | | rs138943062 | 118827194 | GAAAATGGAGGATAAACTGATAAAAGCATA | G | 58.7* | 40 | 32 | 46 | *-* | |
| 6 | | NA | 32860024 | TA | T | 59.8 | 38 | 44 | 53 | *-* | |
| 14 | | rs372935330 | 103722422 | A | AT | 42.8* | 51 | 27 | 25 | *-* | |
| 15 | | rs10643170 | 24392947 | C | CAG | 53.8 | 32 | 23 | 35 | *-* | |
| *Murut (NB12)* | | | | | | | | | |  |  |
| 2 | | NA | 81504200 | ATTC | A | 59.2 | 39 | 30 | 41 | *-* | |
| 5 | | rs1275124302 | 4326975 | C | CCT | 45.2* | 22 | 20 | 21 | *-* | |
| 5 | | NA | 83543889 | CAT | C | 59.9 | 43 | 43 | 55 | *EDIL3* | |
| 5 | | NA | 158946381 | C | CT | 59.8 | 36 | 39 | 37 | *-* | |
| 8 | | rs769885333 | 1206482 | CTG | C | 55.8* | 16 | 19 | 23 | *CTD-2281E23.2* | |
| 12 | | NA | 116677004 | ATATC | A | 59.3 | 41 | 44 | 40 | *MED13L* | |
| 16 | | NA | 83716868 | ATTC | A | 59.6 | 51 | 50 | 44 | *CDH13* | |
| 19 | | rs567942908 | 7484094 | C | CA | 56.1* | 22 | 18 | 17 | *CTD-2207O23.3, ARHGEF18* | |

MQ, mapping quality, indels located in the simple repeats regions (downloaded from http://genome.ucsc.edu/) are indicated with asterisks; DP, read depth; NA, not available.

**Table S13** Summary of CNVs identified in each trio

|  | Bateq | | |  | Mendriq | | |  | Semai | | |  | Dusun | | |  | Murut | | |
| --- | --- | --- | --- | --- | --- | --- | --- | --- | --- | --- | --- | --- | --- | --- | --- | --- | --- | --- | --- |
|  | Total | DUP | DEL |  | Total | DUP | DEL |  | Total | DUP | DEL |  | Total | DUP | DEL |  | Total | DUP | DEL |
| Total CNVs | 1,754 | 334 | 1,420 |  | 2,172 | 465 | 1,707 |  | 1,722 | 267 | 1,455 |  | 1,727 | 303 | 1,424 |  | 1,777 | 313 | 1,464 |
| Recurrent CNVs | 1,339 | 281 | 1,060 |  | 1,469 | 294 | 1,177 |  | 1,301 | 192 | 1,109 |  | 1,356 | 269 | 1,083 |  | 1,426 | 278 | 1,148 |
| Novel CNVs | 390 | 239 | 151 |  | 618 | 388 | 230 |  | 351 | 195 | 156 |  | 325 | 223 | 102 |  | 334 | 231 | 103 |
| Reccurent novel CNVs | 282 | 206 | 76 |  | 283 | 222 | 61 |  | 186 | 132 | 54 |  | 228 | 198 | 30 |  | 235 | 197 | 38 |
| Genic CNVs | 698 | 170 | 528 |  | 952 | 86 | 735 |  | 734 | 151 | 583 |  | 734 | 154 | 580 |  | 714 | 157 | 557 |
| Disrupted genic CNVs | 133 | 84 | 49 |  | 156 | 79 | 76 |  | 143 | 89 | 54 |  | 128 | 75 | 53 |  | 134 | 78 | 56 |

**Table S14** *de novo* CNVs identified in the 5 off-springs

| Chrom-osome | Start | End | Cytoband | Variant Type | Size (bp) | Gene | de novo mutation rate |
| --- | --- | --- | --- | --- | --- | --- | --- |
| *Bateq (BTQ038)* | | | | | | | |
| 1 | 23,908,244 | 23,909,054 | 1p36.11 | DEL | 811 | - | 0.001 |
| 2 | 18,574,244 | 18,574,524 | 2p24.2 | DEL | 281 | - |  |
| *Mendriq (MDQ010)* | | | | | | | |
| 16 | 999,596 | 1,000,584 | 16p13.3 | DEL | 989 | *LMF1* | 0.0005 |
| *Semai (SMI041)* | | | | | | | |
| 3 | 95,116,209 | 95,116,522 | 3q11.2 | DEL | 314 | - | 0.002 |
| 5 | 25,833,357 | 25,833,649 | 5p14.1 | DEL | 293 | - |  |
| 18 | 76,774,376 | 76,777,130 | 18q23 | DEL | 2,755 | - |  |
| *Dusun (NB09)* | | | | | | | |
| 2 | 192,559,415 | 192,559,724 | 2q32.3 | DEL | 310 | - | 0.002 |
| 3 | 28,167,474 | 28,167,782 | 3p24.1 | DEL | 309 | - |  |
| 19 | 350,272 | 351,058 | 19p13.3 | DEL | 787 | - |  |
| 21 | 37,864,450 | 37,864,758 | 21q22.13 | DEL | 309 | *CLDN14* |  |
| *Murut (NB12)* | | | | | | | |
| 5 | 6,571,142 | 6,571,884 | 5p15.31 | DEL | 743 | - | 0.001 |
| 18 | 76,774,376 | 76,777,153 | 18q23 | DEL | 2,778 | - |  |

**Table S15** CNVs sharing across native Malaysian trios

(see Additional file 4.xlsx)

**Table S16** Inheritance of selected genes that known to either lie on the segmental duplication region, or carry CNVs.

| Gene | Population | CN in parents | CN in offspring | Gene | Population | CN in parents | CN in offspring |
| --- | --- | --- | --- | --- | --- | --- | --- |
| *AMY1A/2A* | BTQ | 3 | 3 | DEFB130 | BTQ | 1/3 | 1 |
|  | MDQ | 3 | 3 |  | MDQ | 1 | 1 |
|  | SMI | 3 | 3 |  | SMI | 1 | 1 |
|  | MRT | 3 | 3 |  | MRT | - | - |
|  | DSN | 3 | 3 |  | DSN | 1 | 1 |
| *CCL3L1/CCL4* | BTQ | 1 | 1 | DEFB103A | BTQ | 3 | 3 |
|  | MDQ | - | - |  | MDQ | 3 | 3 |
|  | SMI | 1/4 | 4 |  | SMI | - | - |
|  | MRT | - | - |  | MRT | 1 | 1 |
|  | DSN | - | - |  | DSN | 3 | 3 |
| *CCL3L1/CCL4* | BTQ | 1 | 1 | DEFA1B | BTQ | 4/5 | 4 |
|  | MDQ | - | - |  | MDQ | 4/5 | 5 |
|  | SMI | 1/4 | 4 |  | SMI | - | - |
|  | MRT | - | - |  | MRT | - | - |
|  | DSN | - | - |  | DSN | - | - |
| *FCGR2/3* | BTQ | - | - | LPA | BTQ | 6/7 | - |
|  | MDQ | - | - |  | MDQ | 6/10 | 10 |
|  | SMI | 1/3 | - |  | SMI | 7/9 | 9 |
|  | MRT | - | - |  | MRT | 7/8 | 8 |
|  | DSN | - | - |  | DSN | 4/6 | 4 |
| *GSTM* | BTQ | - | - | CYP2D6 | BTQ | 3 | 3 |
|  | MDQ | 1 | 1 |  | MDQ | - | - |
|  | SMI | 0 | 0 |  | SMI | - | - |
|  | MRT | 0/1 | 1 |  | MRT | 3/4 | 3 |
|  | DSN | - | - |  | DSN | - | - |
| *GSTT* | BTQ | 1 | 1 |  |  |  |  |
|  | MDQ | 1 | 1 |  |  |  |  |
|  | SMI | - | - |  |  |  |  |
|  | MRT | 0 | 0 |  |  |  |  |
|  | DSN | - | 0 |  |  |  |  |

**Table S17** Inherited novel insertions in the 5 native Malaysian trios.

(see Additional file 5.xlsx)
